# Natural variation in the evolutionarily conserved F‐box gene *SDEF* promotes seed dormancy in rice

**DOI:** 10.1111/tpj.71203

**Published:** 2026-10-30

**Authors:** Yuliang Zhou, Yueping Wang, Guangmiao Zhao, Chunbao Liu, Ruizhen Zeng, Wenxuan Liang, Ru Han, Hui Kong, Xiaofan Cao, Minshi Li, Huhui Chen, Bin Tan, Hong Liu, Zhenjiang Xu

**Affiliations:** ^1^ Guangdong Key Laboratory of Plant Molecular Breeding, College of Agriculture South China Agricultural University Guangzhou 510642 China; ^2^ Key Laboratory of Agricultural and Environmental Microbiology, Ministry of Agriculture and Rural Affairs, College of Life Sciences Nanjing Agricultural University Nanjing 210014 China

**Keywords:** *Oryza sativa*, seed dormancy, SCF complex, ABA signaling, CRISPR/Cas9, domestication

## Abstract

Insufficient seed dormancy causes pre‐harvest sprouting (PHS) and severe yield losses in rice; however, genetic resources with practical breeding value remain limited. Here, we isolated a major dormancy gene, *SDEF*, via map‐based cloning using chromosomal single segment substitution lines (SSSLs), identifying a functional SNP (Arg‐to‐Trp) as the causal mutation. CRISPR/Cas9‐mediated knockout of *SDEF* led to PHS susceptibility, while overexpression significantly enhanced dormancy. Importantly, *SDEF* enhanced seed dormancy without compromising grain yield in SSSLs, confirming it as a promising breeding target. Moreover, *SDEF* encodes an angiosperm‐conserved, nuclear‐localized F‐box protein and is preferentially expressed in the embryo, with expression increasing during seed maturation and declining upon germination. Mechanistically, protein interaction assays confirmed that SDEF recruits OSK1, with the Trp‐type SDEF^K^ exhibiting a more stable interaction than the Arg‐type SDEF^H^. *SDEF* is induced by abscisic acid (ABA) and appears to promote seed dormancy through the ABA pathway by activating ABA signaling genes and repressing α‐amylase genes, as evidenced by the reduced ABA sensitivity of *sdef* mutants. Additionally, haplotype analysis revealed that natural variation in *SDEF* is associated with divergent dormancy levels, and reduced nucleotide diversity in cultivated rice indicates that *SDEF* was selected during domestication. Collectively, our results provide insights into the molecular basis of *SDEF‐*mediated dormancy, providing an ideal target for breeding PHS‐resistant rice.

## Significance Statement

Preventing pre‐harvest sprouting in cereals requires enhancing seed dormancy without yield penalties, yet the underlying evolutionary and genetic mechanisms remain largely unresolved. By characterizing the evolutionarily conserved F‐box gene *SDEF*, we reveal how a causal SNP drives natural allelic variation to mechanistically modulate ABA signaling, identifying rare, strong‐dormancy Hap2/Hap5 alleles as immediate, actionable targets for breeding climate‐resilient elite crops.

## INTRODUCTION

Seed germination is a critical step in the plant life cycle. The formation and release of seed dormancy are precise regulatory strategies for germination timing, which are of great significance for the continuation of plant species and agricultural production (Nonogaki, 2019). Dormancy is under negative selection during crop domestication, leading to reduced levels of seed dormancy in cereals, such as rice, making them prone to PHS on the mother plant before harvest. It is estimated that PHS will cause up to $10 billion in economic losses globally each year (Lee et al., 2021) and has a particularly severe impact on the yield and quality of hybrid rice in China. The food security issues caused by pre‐harvest sprouting (PHS) can be overcome by enhancing the level of seed dormancy. Moderate seed dormancy can not only ensure rapid and uniform emergence after sowing but also reduce the occurrence of PHS. Therefore, uncovering natural variations in seed dormancy and deciphering the mechanisms modulating seed dormancy are important goals in rice breeding.

Seed dormancy is a complex trait governed by multiple genes and susceptible to environmental influences. Studies have shown that nearly all plant hormones are involved in the regulation of seed dormancy, with abscisic acid (ABA) and gibberellic acid (GA) being core members of the regulatory network (Tai et al., 2021; Xu et al., 2025). Genetic analysis has identified over 200 QTLs associated with rice seed dormancy and PHS, but only a limited number have been cloned and functionally studied. Among the identified positive regulators, *Sdr4* promotes dormancy by upregulating *OsDOG1‐like* under the modulation of *OsVP1* (Sugimoto et al., 2010; Zhao et al., 2022), while *Rc* and *OsDOG1L‐3* enhance dormancy by promoting ABA biosynthesis and signaling (Gu et al., 2011; Wang et al., 2020). Recently, *QSOXL1* was found to maintain dormancy through the regulation of redox homeostasis (Ma et al., 2026). Conversely, several negative regulators have been elucidated. *SD1* modulates primary dormancy via a GA‐mediated dehydration mechanism (Ye et al., 2015). *SD6* (encoding bHLH108) and *SDR3.1* (encoding MODD) repress seed dormancy by antagonizing ICE2 and inhibiting the transcriptional activity of ABI, respectively (Guo et al., 2024; Xu et al., 2022). Furthermore, *CCT30* has been shown to promote PHS by integrating sugar signaling to suppress the ABA pathway (Fan et al., 2025). Given the extensive utilization of *Rc* and *SD1* in white rice and dwarf breeding, the pool of useful anti‐PHS genes is limited. Consequently, there is an urgent need to clone new seed dormancy genes for rice improvement.

While transcriptional regulation of rice seed dormancy has been well documented, the contribution of post‐translational modulation, such as protein degradation, is emerging as a crucial regulatory node that requires further elucidation. F‐box proteins mediate the degradation of target proteins through the ubiquitin‐26S proteasome system by forming SKP1‐Cullin‐F‐box (SCF) E3 ubiquitin ligases (Ban & Estelle, 2021). This system includes the ubiquitin‐activating enzyme E1, ubiquitin‐conjugating enzyme E2, ubiquitin–ligase E3, and the 26S proteasome. The SCF complex is a major type of E3 ligase, in which F‐box proteins are responsible for substrate recruitment and promote ubiquitination. The F‐box domain at the N‐terminus of F‐box proteins recognizes SKP1 in the SCF complex, while their C‐terminal regions determine substrate specificity. There are 779 F‐box genes in rice (Xu et al., 2009), which are involved in modulating rice growth and development, hormone responses, and biotic and abiotic stresses (Borna et al., 2022; Guo et al., 2021; Ma et al., 2023; Sasaki et al., 2003; Sharma et al., 2023; Sun et al., 2024; Wu et al., 2022). However, the functions of most F‐box genes in rice are still unknown, especially their roles in modulating seed dormancy.

In a previous study, we conducted a comprehensive genetic analysis of rice seed dormancy and identified 25 QTLs associated with seed dormancy. Among them, *qSD7‐1* is a major locus that we fine‐mapped to a 355‐kb interval (Zhou et al., 2017). In this study, we successfully cloned the target gene *SDEF* underlying this major locus using map‐based cloning. Multi‐environment genetic experiments demonstrated that *SDEF* promotes rice seed dormancy. *SDEF* encodes an evolutionarily conserved F‐box protein, which is localized in the nucleus and abundantly expressed in mature seeds. We further confirmed that SDEF interacts with OSK1 (*LOC_Os11g26910*), a member of the SCF‐type E3 ligase, and provide evidence supporting a model in which SDEF may modulate seed dormancy through the ABA signaling pathway. Finally, haplotype analysis indicated that *SDEF* harbors abundant natural variations, suggesting that it can serve as a potential target for enhancing crop resistance against PHS.

## RESULTS

### *qSD7‐1* is a major QTL governing seed dormancy

We previously identified the major dormancy locus *qSD7‐1* on Chromosome 7 using segment substitution lines (SSSLs) in Huajingxian 74 (HJX74) background (Zhou et al., 2017). To clone this gene, we screened two new SSSLs carrying *qSD7‐1*. One SSSL, with a substitution segment of 1.47 Mb flanked by PSM141 and PSM143, was derived from the *japonica* rice variety Khazar, and thus named near‐isogenic line NIL‐K (Figure 1A). Germination tests on freshly harvested seeds or panicles demonstrated that NIL‐K consistently exhibited strong dormancy. Across six growing seasons, the germination rates of NIL‐K were 14, 42, 41, 58, 6, and 39%, respectively, while the control HJX74 had significantly higher rates of 59, 70, 78, 90, 45, and 63%, respectively (Figure 1B–D).

**Figure 1:**
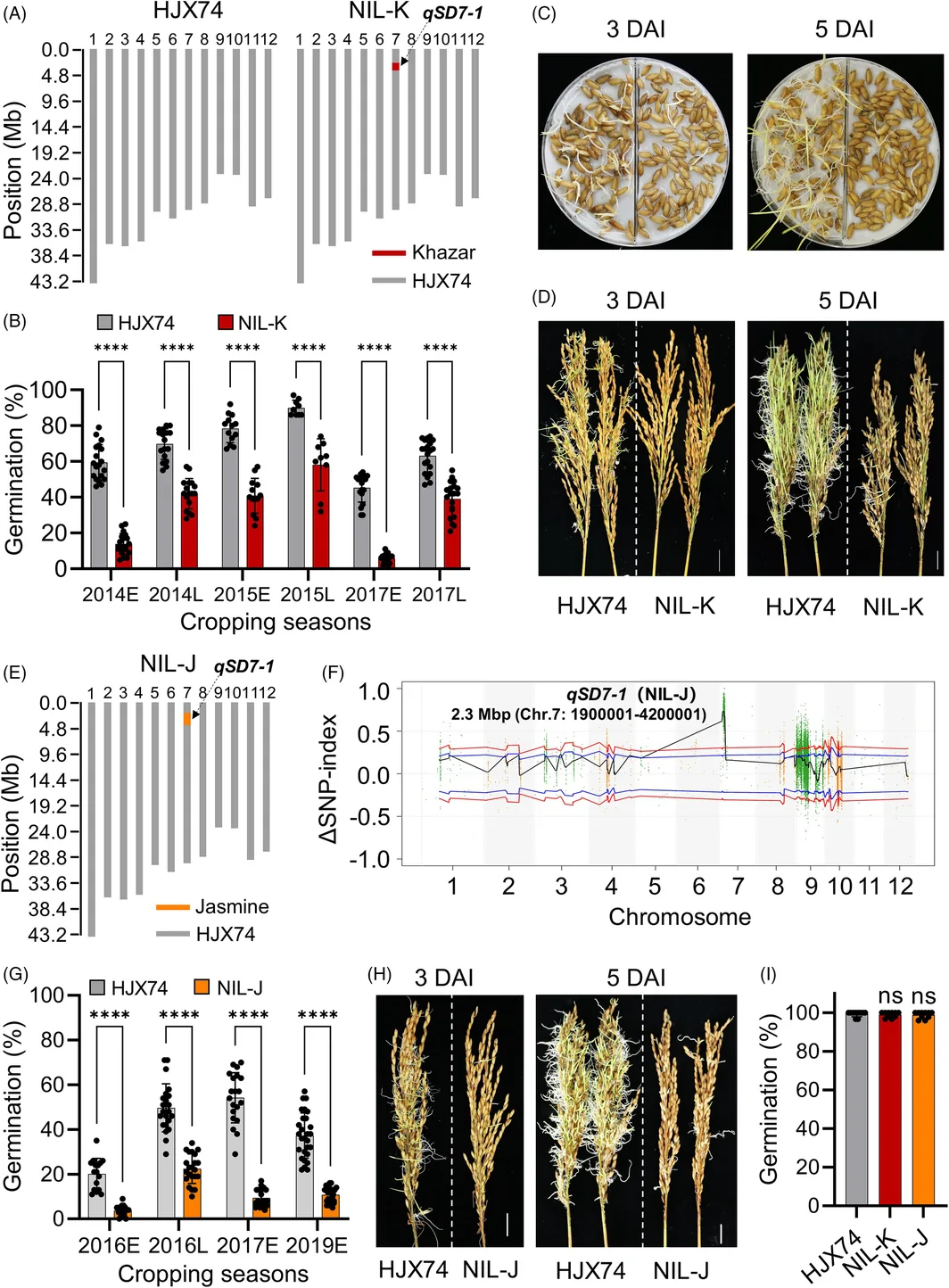
Genotypic and phenotypic characterization of Huajingxian 74 (HJX74), NIL‐K, and NIL‐J. (A) Graphical genotypes of HJX74 and NIL‐K at *qSD7‐1*. NIL‐K is a near‐isogenic line derived from Khazar in the HJX74 background. (B) Germination percentages of freshly harvested seeds or panicles of NIL‐K across six environments. E/L, early/late cropping seasons. (C, D) Representative germination images of freshly harvested seeds (C) and panicles (D) of HJX74 and NIL‐K. DAI, days after imbibition. (E) Graphical genotypes of NIL‐J at *qSD7‐1*. NIL‐J is a near‐isogenic line derived from Jasmine in the HJX74 background. (F) BSA‐seq Manhattan plot for NIL‐J (reference: 9311). Blue and red lines indicate 95% and 99% confidence intervals, respectively; black line shows the fitted ΔSNP‐index. (G) Germination percentages of freshly harvested seeds or panicles of NIL‐J across four environments. (H) Representative germination images of freshly harvested panicles of HJX74 and NIL‐J. (I) Germination percentages of dormancy‐released seeds (50°C, 7 days). *n* = 10. ns, not significant. In (B, G, I), data are means ± SD after 7 days (B, G) or 5 days (I) imbibition. In (B), *n* = 19, 17, 13, 9, 18, 20; In (G), *n* = 16, 22, 20, 28 (ordered by season). ****P < 0.0001, Student's *t*‐test. Scale bars, 2 cm (D, H).

Another SSSL was found through bulk segregant analysis (BSA) sequencing to have several substitution segments, but the locus associated with dormancy was mapped to a 2.3 Mb region on Chromosome 7, ranging from 1.9 to 4.2 Mb. Since this line was derived from the *indica* variety Jasmine, it was thus designated near‐isogenic line NIL‐J (Figure 1E,F). Freshly harvested seeds or panicles of NIL‐J also exhibited strong dormancy, with significantly reduced germination rates (4–23%) compared with HJX74 (20–54%) across four environments (Figure 1G,H). After breaking dormancy, the germination rates of NIL‐K, NIL‐J, and HJX74 all approached 100% (Figure 1I), further confirming that *qSD7‐1* is a key locus governing seed dormancy in rice.

### Map‐based cloning of *SDEF*

To validate *qSD7‐1*, a cross was made between NIL‐K and the recipient parent HJX74, creating an F_2_ population of 200 plants. Germination rates displayed a skewed distribution with most individuals clustering at low germination percentages, and segregation of strong versus weak dormancy fitted a 3:1 ratio (Figure S1a), confirming that *qSD7‐1* functions as a single dominant gene. The NIL‐K allele exhibited an additive effect of −0.22 and a dominance effect of −0.08, accounting for 79.1% of the phenotypic variance. Linkage analysis revealed that *qSD7‐1* was tightly linked to the marker Ui46, with a genetic distance of 0.6 cM (Figure S1b).

To fine‐map *qSD7‐1*, recombinant individuals were selected within the target region, and a large segregating population comprising 3580 individuals was developed for high‐resolution mapping. Using seven newly developed molecular markers, we constructed seven types of homozygous recombinant lines (S1–S7) with shorter substitution segments than NIL‐K. Substitution mapping based on dormancy phenotypes across two growing seasons delimited *qSD7‐1* to a 48.9‐kb physical interval between markers LCB14 and LCB5, which contains nine predicted genes (Figure 2A–D and Table S1). Comparative sequencing of the promoter and coding regions of the candidate genes between NIL‐K and HJX74 revealed variations only in open reading frame (ORF)1 and ORF6. The variation in ORF1 was synonymous. In contrast, ORF6 contained a non‐synonymous substitution at nucleotide position 457 (C‐to‐T), which altered the codon from CGG to TGG. This resulted in an amino acid substitution from arginine (Arg) to tryptophan (Trp) (Figure 2E,F and Figure S2). Consistently, BSA sequencing revealed a single SNP (C‐to‐T) at position 457 in ORF6 between NIL‐J and HJX74, identical to the variant in NIL‐K. Furthermore, gene expression profiling via RiceXPro and quantitative reverse transcription polymerase chain reaction (qRT‐PCR) demonstrated that ORF6 is predominantly expressed in seeds. It displayed the highest expression level of the nine candidate genes in 30‐DAP mature seeds (Figure S3).

**Figure 2:**
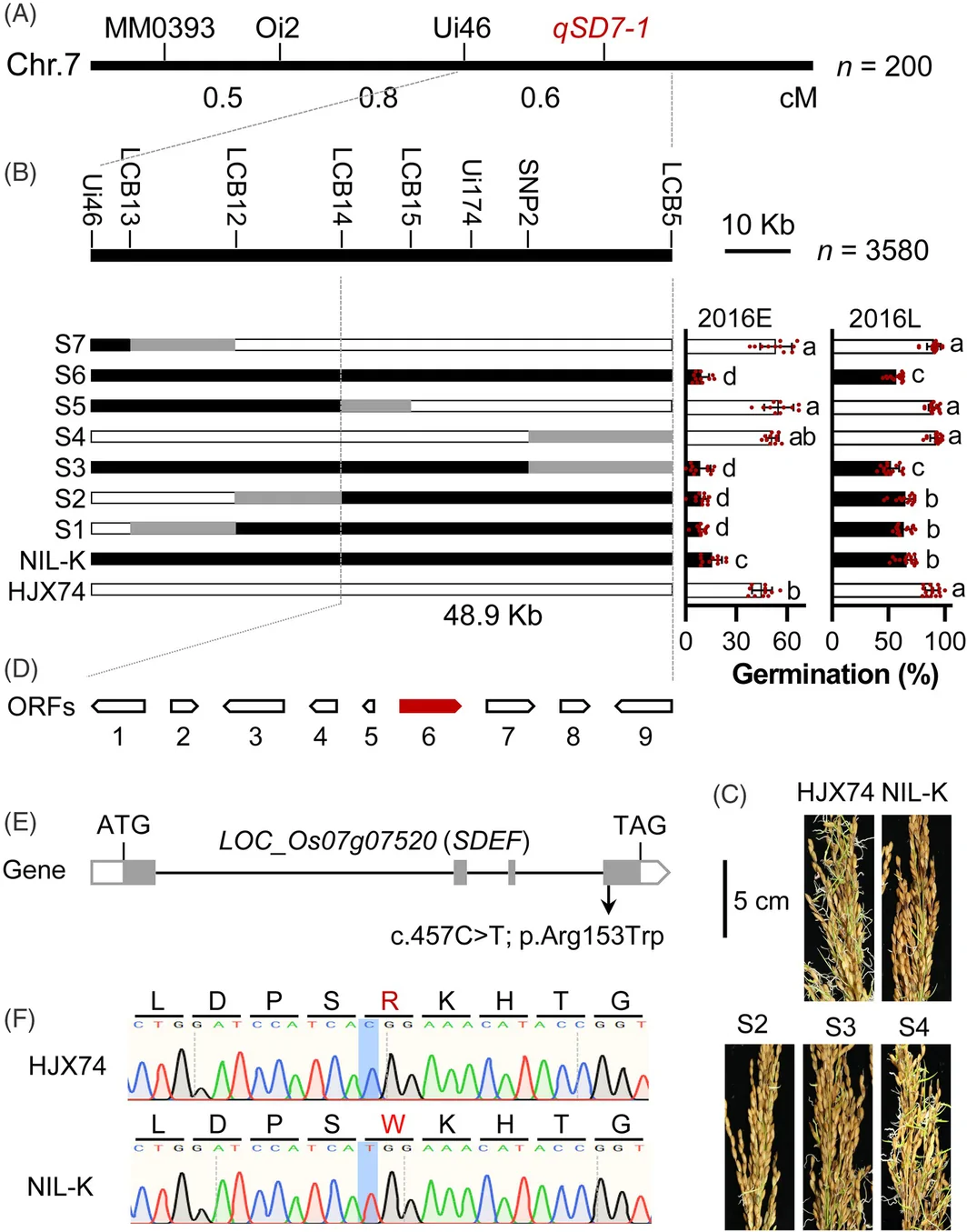
Map‐based cloning of the major seed dormancy locus, *qSD7‐1*. (A) Initial linkage map of *qSD7‐1* on Chromosome 7 based on 200 F_2_ plants. Genetic markers and distances (cM) are shown. Chr, chromosome. (B) Fine mapping of *qSD7‐1*. Seven homozygous recombinants (S1–S7) delimited *qSD7‐1* to a 48.9‐kb region between LCB14 and LCB5 (dotted lines). Black, white, and gray bars represent NIL‐K, Huajingxian 74 (HJX74), and crossover segments, respectively. Germination percentages of freshly harvested panicles are shown on the right (means ± SD; *n* = 15). Different letters denote significant differences (*P* < 0.05, Duncan's multiple‐range test). E/L, early/late cropping seasons. (C) Representative germination images of freshly harvested panicles after 3 days imbibition. (D) Nine predicted open reading frame (ORFs) within the 48.9‐kb region. ORF6 is the candidate gene. (E) Gene structure and sequence variation of *SDEF*. A single‐nucleotide substitution in the fourth exon is indicated. (F) Sequence alignment of *SDEF* between HJX74 and NIL‐K. Red letters (R/W) indicate the amino acid substitution.

Taken together, we identified ORF6 (*LOC_Os07g07520*) as the candidate gene for *qSD7‐1*. This gene encodes the F‐box domain‐containing protein OsFBX217 and was designated as *SDEF* (seed dormancy‐enhancing F‐box gene). The genomic sequence of *SDEF* spans 4897 bp and contains four exons. Its 750‐bp coding sequence (CDS) encodes a protein of 249 amino acids (Table S1).

### *SDEF* promotes seed dormancy

To determine whether *SDEF* affects seed dormancy, CRISPR/Cas9 technology was used to edit *SDEF* in the NIL‐K background, generating three homozygous knockout mutants. Targeting the first exon resulted in 1‐bp, 74‐bp, and 76‐bp deletions, all of which caused frameshift mutations and premature translation termination (Figure 3A,B).

**Figure 3:**
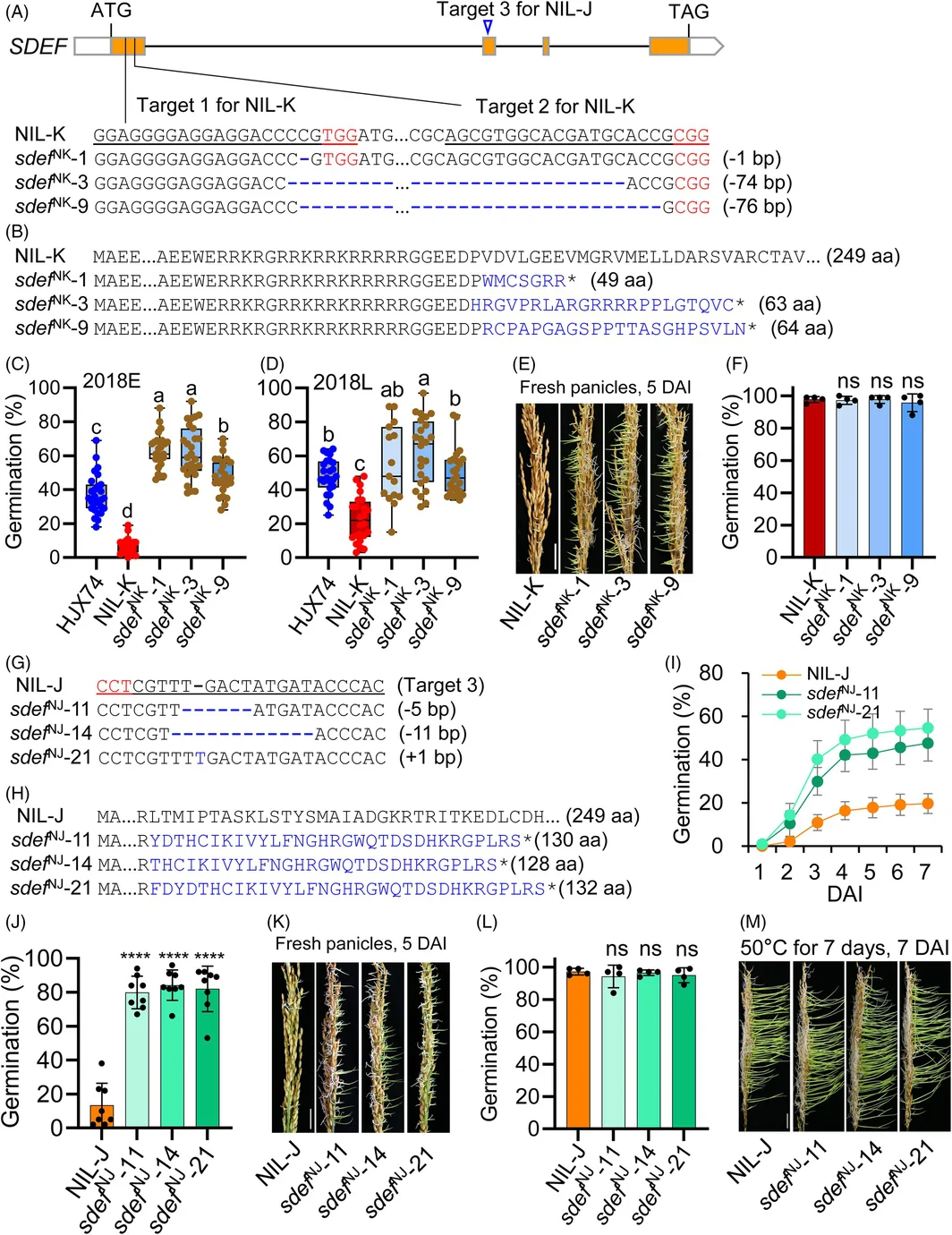
*sdef* mutations decrease seed dormancy. (A) Schematic of *sdef* ^NK^ CRISPR/Cas9 mutations in the NIL‐K background. Targets are underlined; PAMs are red. Blue indicates insertions (letters) or deletions (dashes). (B) Protein sequence alignment of NIL‐K and *sdef* ^NK^. Blue letters, mutated amino acids; *, stop codon. (C, D) Germination percentages of freshly harvested *sdef* ^NK^ panicles (C) and seeds (D) in early (E) and late (L) cropping seasons. Different letters indicate significant differences (*P* < 0.05, Duncan's multiple‐range test). Box plots show medians, interquartile ranges, and minima/maxima (GraphPad Prism 9.2.0). (E) Representative germination images of freshly harvested panicles of *sdef* ^NK^ and NIL‐K. DAI, days after imbibition. (F) Germination percentages of dormancy‐released panicles of *sdef* ^NK^. (G, H), DNA (G) and protein (H) sequence analysis of *sdef* ^NJ^ generated by CRISPR/Cas9 in NIL‐J background. (I) Time‐course germination of freshly harvested *sdef* ^NJ^ seeds (Sanya, 2022E). Data are means ± SD. *n* = 25. (J, L) Germination percentages of freshly harvested (J) and dormancy‐released (L) *sdef* ^NJ^ panicles (Guangzhou, 2022E). ****P < 0.0001, Student's *t*‐test. (K, M) Representative germination images of freshly harvested (K) and dormancy‐released (M) panicles of *sdef* ^NJ^ and NIL‐J. In (C, D, F, J, L), data are means ± SD after 7 days imbibition. *n* = 27 (C), 15–24 (D), 4 (F), 8 (J) and 4 (L). Dormancy was released at 50 °C for 7 days (F, L, M). Scale bars, 2 cm (E, K, M). ns, not significant.

In two growing seasons, freshly harvested mature panicles and seeds from *SDEF* knockout mutants (*sdef* ^NK^‐1, *sdef* ^NK^‐3, and *sdef* ^NK^‐9) exhibited significantly higher germination rates after 7 days of imbibition compared with the control NIL‐K (Figure 3C–E). For example, in the early cropping season of 2018, the germination rates of *sdef* ^NK^‐1, *sdef* ^NK^‐3, and *sdef* ^NK^‐9 were 63%, 62%, and 48%, respectively, while the germination rate of NIL‐K was only 6% (Figure 3C). After dormancy release, the knockout mutants showed germination rates comparable to NIL‐K (Figure 3F), indicating that the observed differences were due to seed dormancy rather than seed vigor. In addition, the *sdef* ^NK^ mutants exhibited higher germination rates than HJX74 (Figure 3C,D), suggesting that the *SDEF* allele in HJX74 is a weak rather than a null allele.

Similarly, targeting the *SDEF* second exon in NIL‐J generated *sdef* ^NJ^ mutants with 5‐ and 11‐bp deletions and a 1‐bp insertion, all causing frameshifts and protein truncation (Figure 3G,H). In both Sanya and Guangzhou, the mutant lines exhibited higher germination rates compared with that of control NIL‐J, while after dormancy release, both showed similar good germination performance (Figure 3I–M), indicating that *SDEF* mutations result in reduced seed dormancy. Furthermore, we overexpressed the coding region of *SDEF* from NIL‐K in HJX74, obtaining three homozygous overexpression lines with elevated expression levels (*SDEF* ^K^‐OE1, *SDEF* ^K^‐OE2, and *SDEF* ^K^‐OE3) (Figure 4A). After breaking dormancy at 50°C for 7 days, the overexpression lines and HJX74 showed similar high germination rates (Figure 4B,C). However, in five different growing seasons, freshly harvested overexpression lines exhibited significantly higher seed dormancy levels, with germination rates lower than those of HJX74 (Figure 4D,E).

**Figure 4:**
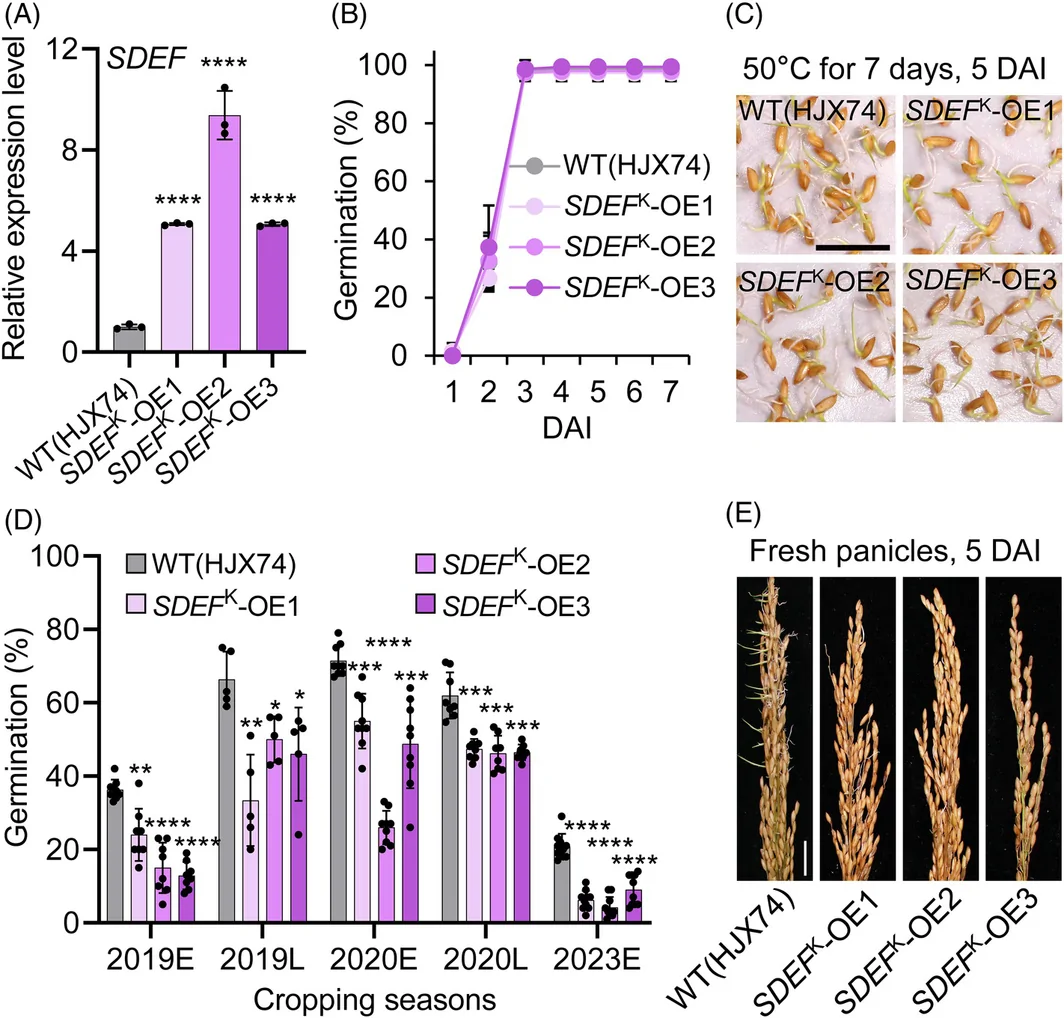
*SDEF* overexpression enhances seed dormancy. (A) *SDEF* expression in Huajingxian 74 (HJX74) and overexpression (OE) lines determined by quantitative reverse transcription polymerase chain reaction (qRT‐PCR). *OsActin1* was used as an internal control. Data are means ± SD (*n* = 3). (B, C) Germination time‐course (B) and representative images (C) of dormancy‐released seeds (50°C, 7 days). DAI, days after imbibition; *n* = 15 (HJX74) and 5 (OE). (D, E) Germination percentages (D) and representative images (E) of freshly harvested panicles across five environments. E/L, early/late cropping seasons. Data are means ± SD after 7 days imbibition. *n* = 8, 5, 9, 8, 10 (ordered by season). In (A, D), *P < 0.05, **P < 0.01, ***P < 0.001, ****P < 0.0001. Student's *t*‐test. In (C, E), Scale bars, 2 cm.

In summary, these results confirm that *SDEF* is the causal gene of *qSD7‐1* and functions as a positive regulator of seed dormancy in rice. To assess the breeding value of *SDEF*, we also evaluated the agronomic traits of NIL‐K and NIL‐J. Compared with HJX74, both NIL‐K and NIL‐J exhibited increased plant height and higher grain number per panicle, while grain length and 1000‐grain weight were reduced. However, the final single‐plant yield did not show significant differences (Figure S4).

### *SDEF* is predominantly expressed in embryos

Protein sequence analysis revealed that SDEF contains an F‐box‐like domain located at the N‐terminal region, spanning residues 48–83 (Figure S5a). The N‐terminal region of SDEF and its homologs is highly variable, whereas the F‐box‐like domain and the C‐terminal region are highly conserved (Figures S5b and S6). Multiple sequence alignment showed that SDEF shares over 80% identity with orthologs from Poaceae, such as *Panicum virgatum* (XP_039784084.1, 80.8%), *Triticum aestivum* (XP_044395551.1, 82.7%), and *Aegilops tauschii* (XP_020165376.1, 82.7%) (Figure S6). Phylogenetic analysis revealed that SDEF homologs are evolutionarily conserved across angiosperms, with particularly high conservation in Poaceae, and lack close paralogs in the rice F‐box superfamily (Figure S7).

RiceXPro data revealed broad *SDEF* expression in rice tissues, predominantly in the embryo (Figure S3a). To further investigate *SDEF* expression, we performed qRT‐PCR analysis on seeds from NIL‐K, NIL‐J, and HJX74. The results revealed that *SDEF* expression level increased gradually throughout seed development, peaking at 20 DAP (Figure 5A). A similar expression trend was observed in the isolated embryos (Figure 5B). Although differences in *SDEF* expression between NIL‐K and HJX74 were detected during the late stages of seed development, no distinct pattern emerged (Figure 5A,B). Notably, during the first 48 h of seed imbibition, *SDEF* expression in HJX74 progressively declined, while, in contrast, it remained high or even increased in the seeds of NIL‐J (Figure 5C) and in the embryos of NIL‐K (Figure 5D).

**Figure 5:**
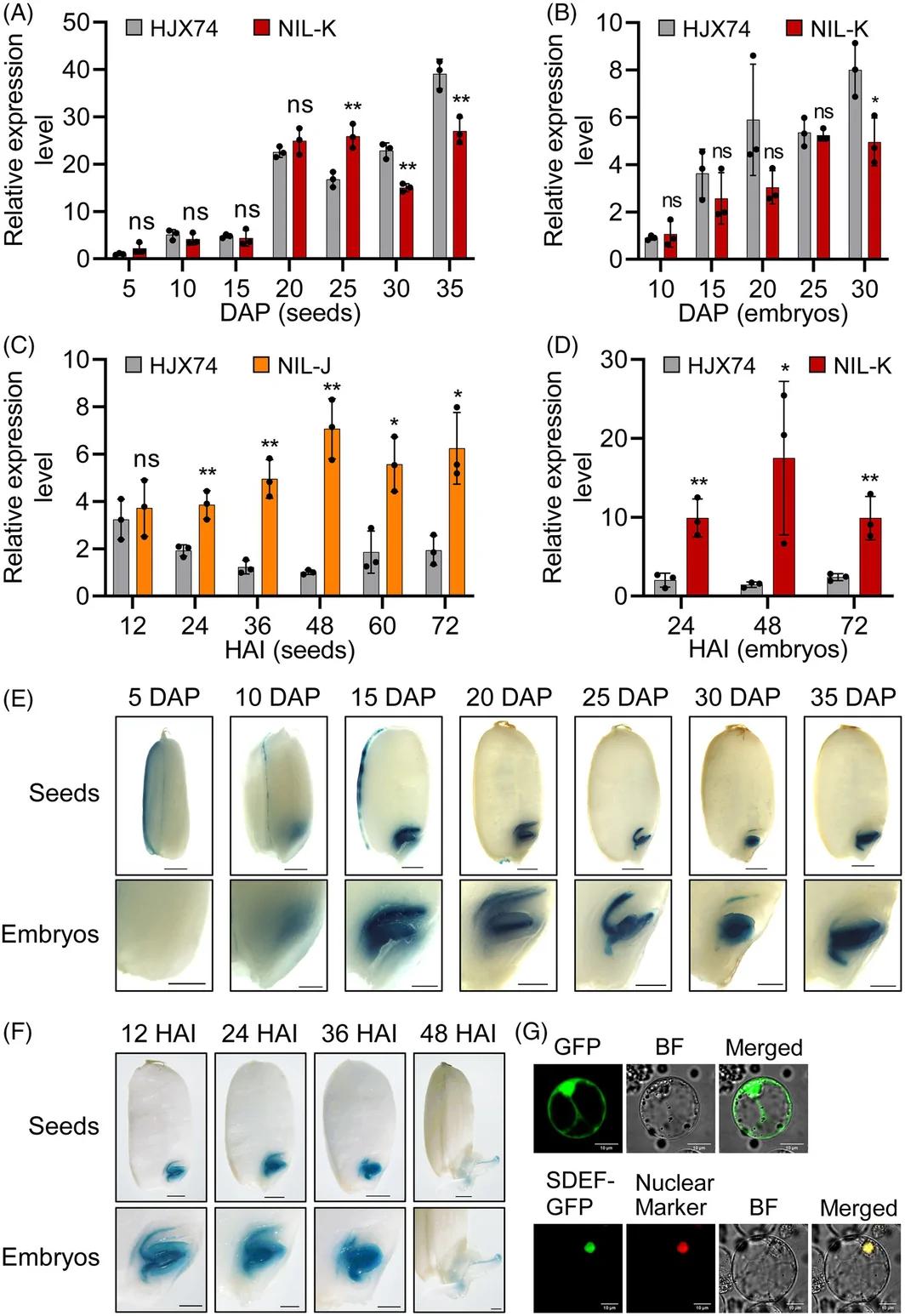
Expression pattern of *SDEF* and its protein subcellular localization. (A–D) Relative expression of *SDEF* in developing seeds (A), developing embryos (B), imbibed seeds (C), and imbibed embryos (D). Transcript levels were assessed by qRT‐PCR using *OsActin1* as an internal control. Data are means ± SD (*n* = 3). *P < 0.05, **P < 0.01, Student's *t*‐test. ns, not significant. DAP, days after pollination; HAI, hours after imbibition. (E, F) *pSDEF::GUS* staining in developing (E) and imbibed (F) seeds. Longitudinal sections of seeds are shown with embryos magnified. Scale bars, 1 mm (seeds) and 0.5 mm (embryos). (G) Subcellular localization of SDEF‐GFP in rice protoplasts. BF, bright field. Scale bars, 10 μm.

Consistent with qRT‐PCR results, GUS staining of *pSDEF::GUS* transgenic plants confirmed *SDEF* expression in developing and imbibed seeds (Figure 5E,F). GUS signals were initially (5–15 DAP) found in embryos and vascular bundles but later (20–35 DAP) confined to the embryos, especially the plumules (Figure 5E). During imbibition, GUS activity was also predominantly localized in the embryos, but the signal gradually weakened as germination progressed (Figure 5F). Additionally, GUS staining was observed in the roots, stems, and leaves, concentrated mainly in the vascular bundles (Figure S8). Collectively, these findings confirm the robust expression of *SDEF* in seeds, particularly in the embryos, suggesting a potential role in modulating seed dormancy. Moreover, subcellular localization analysis showed that SDEF‐GFP co‐localized with a nuclear marker in rice protoplasts, indicating that SDEF is localized in the nucleus (Figure 5G).

### SDEF interacts with OSK1, a component of the SCF‐type E3 ubiquitin ligase

Given that F‐box proteins interact with SKP1 to form SCF complexes, and SDEF contains a predicted F‐box domain, we investigated the interaction between SDEF and OSK1 (a rice SKP1 homolog) using yeast two‐hybrid (Y2H) assays (Figure 6A). Both full‐length SDEF (SDEF^K^/SDEF^H^) and the N‐terminal fragment (SDEF‐NF) interacted with OSK1. Interestingly, the deletion mutant SDEF^K^‐ΔNF interacted with OSK1, whereas SDEF^H^‐ΔNF did not (Figure 6B). Additionally, while OSK1 interacted with Cullin, no direct interaction was observed between SDEF and Cullin (Figure S9), supporting the canonical SCF assembly model. Moreover, a pull‐down assay confirmed that MBP‐OSK1, but not MBP alone, specifically bound His‐SDEF^K^ (Figure 6C), demonstrating a direct in vitro interaction between SDEF and OSK1.

**Figure 6:**
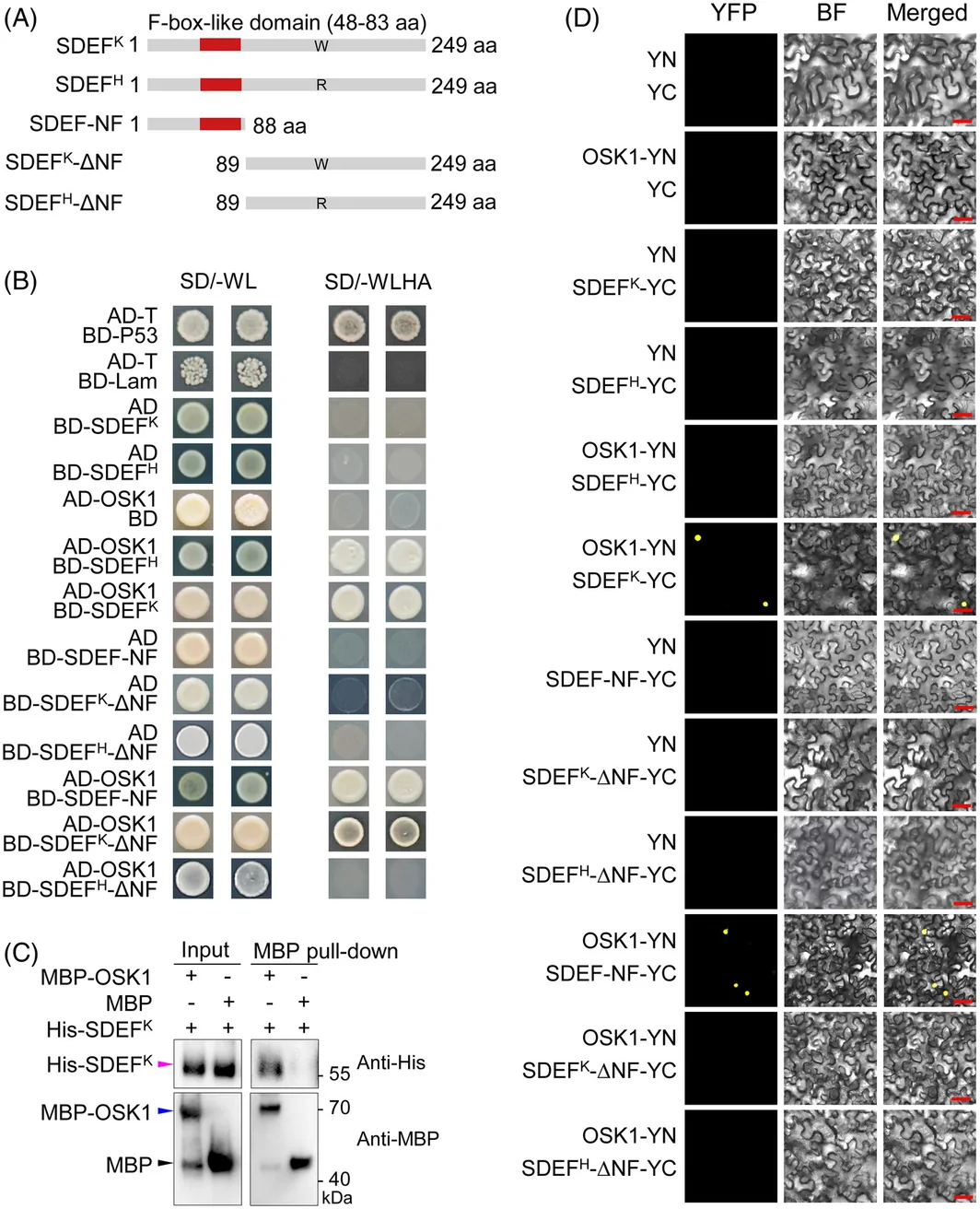
SDEF interacts with OSK1. (A) Schematic of the full length and truncated SDEF proteins used in yeast two‐hybrid (Y2H) and bimolecular fluorescence complementary (BiFC) assays. SDEF^K^ and SDEF^H^ denote the SDEF from Khazar and Huajingxian 74 (HJX74), respectively. W and R indicate the mutated amino acids. (B) Y2H assays showing interaction of SDEF and OSK1. Yeast transformants were grown on control SD‐WL (‐Trp/‐Leu) or selective SD‐WLHA (‐Trp/‐Leu/‐His/‐Ade) media. AD‐T/BD‐Lam, negative control; AD‐T/BD‐P53, positive control. (C) In vitro MBP pull‐down assay of the interaction between SDEF and OSK1. Immunoblot analyses were performed using anti‐His or anti‐MBP antibodies. The experiment was repeated three times with similar results. (D) BiFC visualization of SDEF‐OSK1 interaction in *N. benthamiana* leaves. Constructs expressing N‐terminal (YN) and C‐terminal (YC) YFP fusions were co‐infiltrated. YFP fluorescence indicates interaction. BF, bright field. Scale bar, 50 μm.

We further confirmed the SDEF‐OSK1 interaction using bimolecular fluorescence complementary (BiFC) assays in *Nicotiana benthamiana*. Upon co‐expression of SDEF‐YC and OSK1‐YN, strong fluorescence was observed for the full‐length SDEF^K^ and SDEF‐NF. In contrast to the Y2H results, neither SDEF^H^ nor SDEF^K^‐ΔNF produced a visible BiFC signal (Figure 6D). This discrepancy suggests that the interactions observed in yeast for SDEF^H^ and SDEF^K^‐ΔNF may be unstable in plant cells. These findings support the conclusion that the F‐box domain is essential for the stable interaction between SDEF and OSK1.

### *SDEF* promotes seed dormancy by enhancing ABA signaling pathway

To explore the molecular basis of *SDEF*‐mediated seed dormancy, transcriptome analysis of 24 h‐imbibed seeds was conducted. A total of 146 up‐regulated and 149 downregulated genes were identified in NIL‐J compared with HJX74 (Figure 7A and Table S2). Interestingly, genes involved in ABA signaling, including *OsVP1*, *OsbZIP10*, *Sdr4*, and *OsMFT2*, showed markedly higher expression, whereas α‐amylase genes exhibited reduced transcript levels (Figure 7B). These expression patterns were further confirmed by qRT‐PCR (Figure 7C).

**Figure 7:**
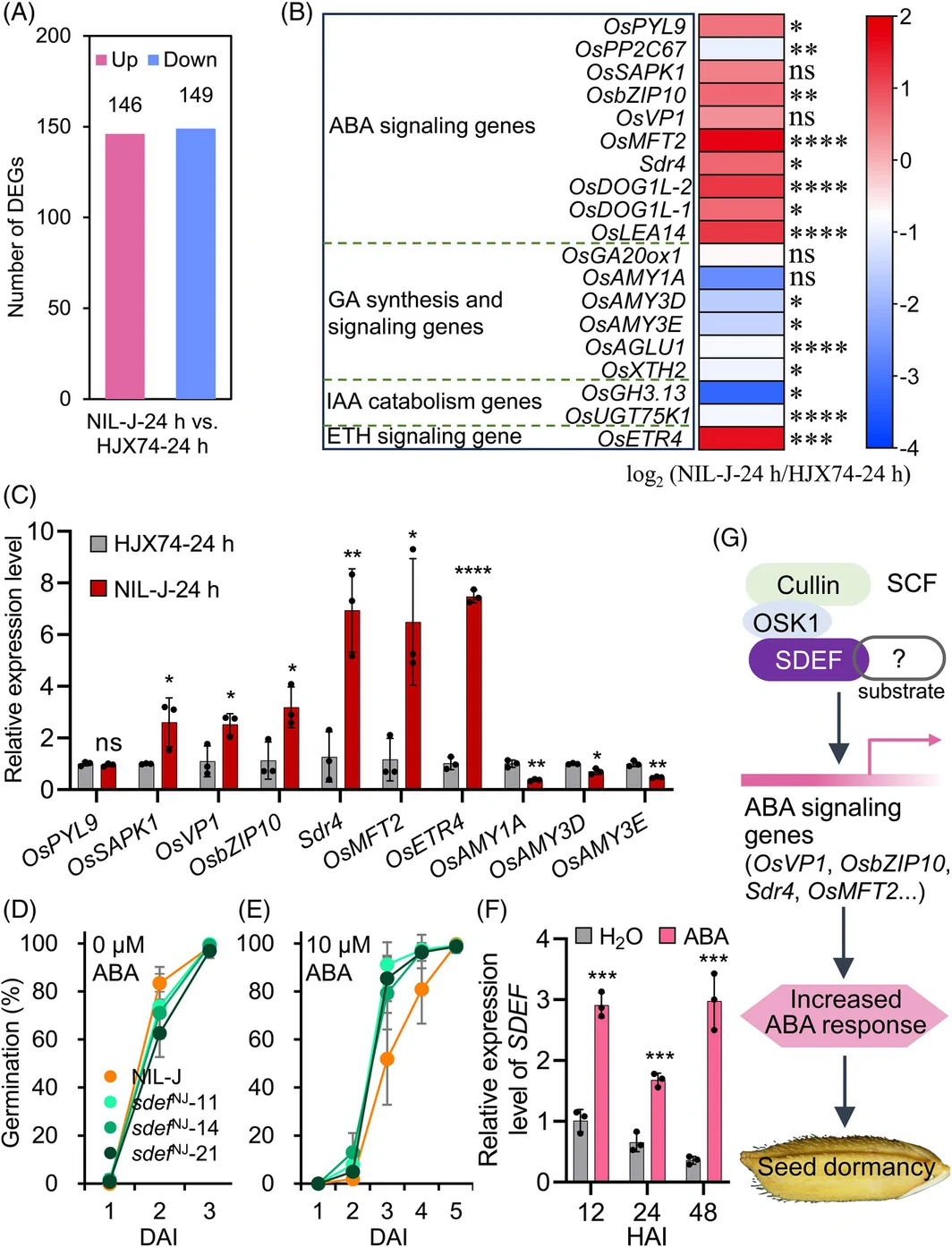
*SDEF* enhances the abscisic acid (ABA) signaling pathway. (A) Differentially expressed genes (DEGs) between NIL‐J and Huajingxian 74 (HJX74) seeds imbibed for 24 h. (B) DEGs involved in plant hormone metabolism and signaling. *FDR <0.05, **FDR <0.01, ***FDR <0.001, ****FDR <0.0001. FDR, false discovery rate. (C) Relative expression of ABA and gibberellic acid (GA) signaling genes in 24 h‐imbibed seeds between NIL‐J and HJX74. (D, E) Germination of NIL‐J and *sdef* ^NJ^ mutants under normal and ABA‐treated conditions. DAI, days after imbibition. (F) *SDEF* expression in response to 15 μM ABA during seed imbibition in HJX74. HAI, hours after imbibition. (G) Proposed model for *SDEF*‐mediated seed dormancy regulation. Data are means ± SD (*n* = 3 in C, F; *n* = 9 in D, E). In (C, F), transcript levels were assessed by qRT‐PCR using *OsActin1* as an internal control. *P < 0.05, **P < 0.01, ***P < 0.001, ****P < 0.0001. ns, not significant. Student's *t*‐test.

In addition, endogenous hormone analysis showed that ABA content was higher in NIL‐K than in HJX74 at 20 DAP. Interestingly, IAA levels remained consistently elevated in NIL‐K from 10 to 30 DAP (Figure S10), suggesting potential IAA‐ABA interaction in dormancy regulation. Moreover, dormancy‐released seeds of NIL‐J and *sdef* ^NJ^ showed different responses to exogenous ABA. Under 10 μM ABA, seed germination of NIL‐J was more strongly inhibited than that of *sdef* ^NJ^ (Figure 7D,E). Consistently, the expression of *SDEF* was significantly induced by 15 μM ABA during imbibition (Figure 7F).

Taken together, the combined results support a model in which *SDEF* promotes seed dormancy by reinforcing ABA signaling during germination. *SDEF* functions as part of an SCF‐type E3 ubiquitin–ligase complex with OSK1 and Cullin, potentially mediating the ubiquitination of unidentified substrates to amplify ABA responses (Figure 7G).

### Natural variations of *SDEF* are associated with seed dormancy

To explore the natural variation of *SDEF* and identify favorable alleles, haplotype analysis was performed using the 3K rice genome database. Six single‐nucleotide polymorphisms (SNPs) located in the 1‐kb promoter and coding regions defined five major haplotypes (Table S3). Hap1 was the predominant haplotype, comprising 2088 accessions across all ten rice subpopulations, whereas Hap2–Hap5 were rare haplotypes (Figure 8A,B).

**Figure 8:**
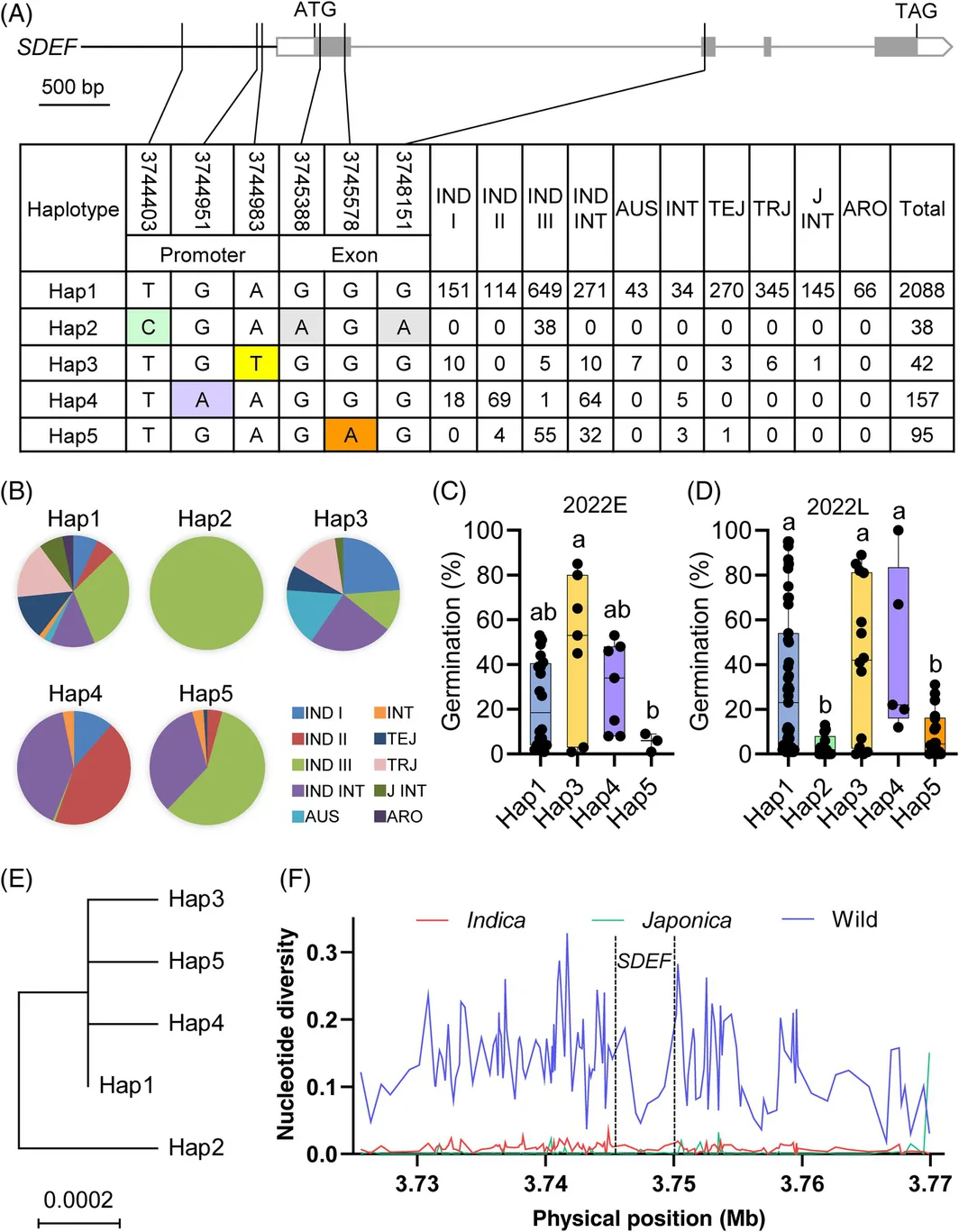
Haplotype analysis of *SDEF*. (A) Gene structure and haplotypes of *SDEF* across the 1‐kb promoter and coding region in 3K rice panel. Nipponbare (Hap1) is the reference allele. Color letters indicate alternative nucleotides, while gray letters indicate synonymous substitutions. IND, *indica*; INT, intermediate; AUS, *aus*; TEJ, temperate *japonica*; TRJ, tropical *japonica*; ARO, aromatic. (B) Subpopulation distribution across the five *SDEF* haplotypes. (C, D) Germination percentages of freshly harvested panicles for the five *SDEF* haplotypes. E/L, early/late cropping seasons. Data are means ± SE after 7 days imbibition. 2022E, *n* = 20 (Hap1), 7 (Hap3 and Hap4), and 3 (Hap5). 2022L, *n* = 43 (Hap1), 13 (Hap2), 14 (Hap3), 5 (Hap4), and 18 (Hap5). Each accession contains 10 independent plants. Different letters denote significant differences (*P* < 0.05, Duncan's multiple‐range test). (E) Phylogenetic tree of the five *SDEF* haplotypes. Scale bar indicates substitutions per site. (F) Nucleotide diversity of *SDEF* in cultivated and wild rice. Dotted vertical lines indicate the *SDEF* genomic region.

Germination assays revealed significant differences in seed dormancy among haplotypes. Hap1, Hap3, and Hap4 exhibited relatively weak dormancy, with mean germination rates of 32%, 42%, and 44% (2022L), respectively. In contrast, Hap2 and Hap5 showed stronger dormancy, with mean germination rates of 4% and 9%, respectively (Figure 8C,D). At the subpopulation level, IND I, TEJ, and TRJ displayed weaker dormancy, whereas IND II, IND III, IND INT, AUS, and INT exhibited stronger dormancy (Figure S11). In addition, subpopulation‐matched comparisons within IND III and IND INT revealed that Hap2 and Hap5 consistently showed significantly lower germination rates than Hap3 within the same subpopulations (Figure S12). Notably, the SNP at position 457 (C>T) in *SDEF* coding region was excluded from haplotyping due to the rare C allele, present in 11 accessions. These accessions (457‐C), which are identical to Hap1 (457‐T) at all other SNPs, exhibited no significant dormancy difference from Hap1 (Figure S13), likely reflecting diverse genetic background effects of natural accessions.

Phylogenetic analysis showed that Hap1, Hap3, Hap4, and Hap5 were closely related, whereas Hap2 was divergent (Figure 8E). Nucleotide diversity (π) across the *SDEF* locus and its 40‐kb flanking regions was severely reduced in cultivated rice compared with wild rice. The average π decreased from 0.1399 in wild rice to 0.0094 in *indica* and 0.0030 in *japonica*. This drastic loss of genetic diversity (>14‐fold reduction) strongly supports a selective sweep during domestication (Figure 8F).

## DISCUSSION

With increasing climate volatility, stable crop production faces growing challenges. Seed dormancy is a key agronomic trait balancing environmental adaptation and yield stability (Jørgensen et al., 2026; Nonogaki, 2019), yet domestication‐driven loss of dormancy alleles has increased the risk of PHS in rice and other cereals (Xu et al., 2025). To address the elusive molecular basis underlying natural variation in seed dormancy, we identified *SDEF*, a highly conserved gene across angiosperms, via map‐based cloning. We systematically characterized its natural variation, molecular function, regulatory network, and selection signatures, providing valuable insights into the regulation of rice seed dormancy.

Although rice contains numerous F‐box genes, most remain functionally uncharacterized (Xu et al., 2009). *SDEF* was identified from two NILs, NIL‐K and NIL‐J, derived from the *japonica* cultivar Khazar and the *indica* cultivar Jasmine, respectively. Both lines exhibited significantly stronger seed dormancy than the recurrent parent HJX74 under multiple environments (Figure 1). A causal SNP (R153W) was identified as the determinant of this phenotypic divergence (Figure 2E,F and Figure S2). Similar to *QSOXL1* (Ma et al., 2026), *GL4* (Wu et al., 2017), and *qSH1* (Konishi et al., 2006), the R153W substitution in *SDEF* underscores the pivotal role of SNP‐driven genetic variations in shaping key agronomic traits.

Functional analyses demonstrated *SDEF* as a positive regulator of seed dormancy: loss‐of‐function mutations caused PHS susceptibility (Figure 3), whereas overexpression enhanced dormancy (Figure 4). Previous studies have shown that unfavorable pleiotropy is common in dormancy genes. For instance, *Rc* affects pericarp color (Gu et al., 2011), *SD1* plant height (Ye et al., 2015), *CCT30* heading date (Fan et al., 2025), *OsFIE1* grain quality (Cheng et al., 2025), and *SDR3.1* drought tolerance (Guo et al., 2024). Notably, although *SDEF* alleles in NIL‐K and NIL‐J slightly reduced grain size, increased grain number compensated this effect (Figure S4), indicating that *SDEF* enhances seed dormancy without a yield penalty and is a promising breeding target. Unlike most rice dormancy genes, *SDEF* encodes an angiosperm‐conserved F‐box protein lacking paralogs among over 700 rice F‐box genes (Figure S7), suggesting it is a unique and essential regulator of rice seed dormancy.

F‐box proteins are core components of the SCF E3 ubiquitin–ligase complex (Zhang et al., 2019). F‐box‐mediated substrate degradation is a conserved mechanism modulating hormone signaling and seed germination. Several F‐box proteins modulate germination via distinct hormone pathways, including RIFP1 in ABA signaling (Li et al., 2016), TIR1 in auxin signaling (Guo et al., 2021), MAX2 in karrikin signaling (Nelson et al., 2011), and OsFBK12 in ethylene signaling (Chen et al., 2013). Consistent with this, SDEF localizes to the nucleus and interacts with OSK1 (Figure 6), indicating that it may function in an SCF complex to modulate seed dormancy. Notably, the strong‐dormancy allele SDEF^K^ interacts robustly with OSK1, whereas the weak‐dormancy allele SDEF^H^ does not (Figure 6). These results suggest that the R153W mutation strengthens SDEF‐OSK1 assembly, which may subsequently enhance seed dormancy via the SCF complex.

ABA is the central hormone governing the establishment and maintenance of seed dormancy (Sajeev et al., 2024; Xu et al., 2025). Our transcriptome data indicate that *SDEF* does not function as a regulator of ABA biosynthesis but is more likely a critical node within the ABA signaling pathway. In the strong‐dormancy line NIL‐J, key positive regulators of ABA signaling—such as *OsVP1*, *OsbZIP10*, *Sdr4*, and *OsMFT2*—were markedly upregulated (Figure 7B,C), all of which have been demonstrated to promote seed dormancy (Bhatnagar et al., 2017; Chen et al., 2021; Shen et al., 2024; Sugimoto et al., 2010). Conversely, several downstream α‐amylase genes (*OsAmy1A*, *OsAmy3D*, and *OsAmy3E*) that facilitate germination were strongly repressed (Figure 7B,C). Moreover, *sdef* ^NJ^ mutants exhibited reduced sensitivity to ABA (Figure 7D,E), whereas exogenous ABA induced *SDEF* expression (Figure 7F), implying that *SDEF* may be involved in a positive feedback regulation of ABA signaling. While the ABA/GA balance is central to dormancy, the strong‐dormancy NIL‐K also exhibited elevated IAA levels during seed development (Figure S10). Given that IAA is known to reinforce ABA signaling (Huang et al., 2025; Liu et al., 2013; Mei et al., 2023), *SDEF* likely functions as a hub integrating these signals to modulate seed dormancy. Based on these findings, we propose a regulatory model for SDEF: SDEF assembles with OSK1 and Cullin to form the SCF^SDEF^ ubiquitin–ligase complex, which may modulate seed dormancy by influencing ABA signaling and starch‐metabolism pathways via ubiquitin‐mediated turnover of unidentified substrates (Figure 7G).

Crop domestication is often associated with reduced seed dormancy to ensure uniform germination (Purugganan & Fuller, 2009). Haplotype analysis showed that Hap1 is the predominant *SDEF* haplotype and is associated with relatively weak dormancy at the population level, consistent with artificial selection for rapid and uniform germination during rice domestication (Figure 8A–D). Similar domestication‐driven reductions in dormancy have been reported for other rice genes, including *Sdr4*, *Rc*, *SDR3.1*, and *SD6* (Gu et al., 2011; Guo et al., 2024; Sugimoto et al., 2010; Xu et al., 2022). Conversely, the rare strong‐dormancy alleles Hap2 and Hap5 represent valuable genetic resources for improving resistance to PHS. Despite directional selection at the *SDEF* locus, *indica* rice retains higher haplotype diversity than *japonica* (Figure 8A,B). It may partly reflect the generally higher genome‐wide diversity of *indica* in 3K rice panel (3K RGP, 2014); however, the differential selection may have also shaped this locus. *Japonica* is nearly fixed for the weak‐dormancy Hap1, an adaptation to cold and dry temperate regions, whereas *indica* retains diverse haplotypes, including Hap2 and Hap5, potentially suited to warm and humid tropical and subtropical environments. This environmental shaping of dormancy alleles parallels recent findings at the barley *MKK3* locus (Jørgensen et al., 2026). Furthermore, the detection of a pronounced selective sweep at the *SDEF* locus identifies it as a major target of domestication (Figure 8F). Thus, the evolution of *SDEF* likely reflects a balance between human‐mediated selection and ecological adaptation.

Although Hap1 accessions harbor the same 457‐T allele found in NIL‐K and NIL‐J, only a subset of them exhibit strong seed dormancy (Figure 8C,D). We hypothesize that the phenotypic effect of *SDEF* may be enhanced or masked by epistatic interactions with other dormancy genes (such as *Sdr4*, *SD6*, or *SDR3.1*) that vary across subpopulations. This underscores the value of our NIL strategy: by fixing the genetic background, we isolated the *bona fide* effect of *SDEF*, which is otherwise diluted in the diverse 3K panel. While *SDEF* remains a valuable breeding target, its background‐dependent effect suggests that future breeding should prioritize multi‐gene pyramiding to achieve stable PHS resistance.

In conclusion, *SDEF* is an angiosperm‐conserved F‐box gene selected during domestication that is proposed to promote seed dormancy via the SCF^SDEF^ complex, likely by coordinating ABA signaling and starch mobilization. These results provide insights into a distinct dormancy mechanism and highlight *SDEF* as a valuable target for breeding PHS‐resistant varieties. Future work focusing on identifying SCF^SDEF^ substrates and elucidating *SDEF*‐mediated IAA‐ABA interaction will further advance our understanding of seed dormancy regulation and guide crop improvement strategies.

## MATERIALS AND METHODS

### Plant materials and cultivation

Khazar (*japonica*) and Jasmine (*indica*) were used as donor parents, and HJX74 (*indica*) as the recurrent parent to construct SSSLs (Zhang, 2021). Two near‐isogenic lines, NIL‐K and NIL‐J, were developed using marker‐assisted selection in the HJX74 background. The *sdef* mutants were generated in the NIL‐K and NIL‐J backgrounds using CRISPR/Cas9, and *SDEF* overexpression lines were created in the HJX74 background via *Agrobacterium*‐mediated transformation. Rice germplasms for haplotype analysis were acquired from the Chinese Academy of Agricultural Sciences and College of Agriculture, South China Agricultural University. All rice materials were grown under field conditions in Guangzhou and Sanya, China. Mature seeds were harvested for subsequent experiments.

### Evaluation of seed dormancy

Seed dormancy was evaluated following Zhou et al. (2017), with minor modifications. Plants were tagged daily at heading, and fresh seeds or panicles were harvested at approximately 35 DAP from synchronously heading individuals. Seeds were placed in 9‐cm Petri dishes containing two layers of moist filter paper and incubated in sealed plastic boxes (32 × 23 × 4.5 cm). Panicles were directly placed in sealed plastic boxes with two layers of moist filter paper and covered with an additional layer of moist filter paper. The chamber was set at 30°C with 16 h light/8 h dark. Germination, defined as ≥1 mm radicle protrusion, was recorded on 3, 5, and 7 days. Meanwhile, a control subset was dried at 50°C for 7 days to break dormancy and confirm seed vigor (Zhou et al., 2017).

### Bulk segregant analysis sequencing

BSA sequencing was performed as described previously (Pan et al., 2021) with minor modification. Briefly, NIL‐J was backcrossed with HJX74 to produce an F_2_ population of 312 plants. Young leaves were individually collected and stored at −80°C. Equal‐sized leaf samples from 25 plants exhibiting extremely strong or weak seed dormancy were bulked into SD‐pool and WT‐pool, respectively. These pools and the parents were sequenced to 30× depth using the Illumina platform (Genedenovo, Guangzhou, China). Clean reads were mapped to the *indica* 9311 reference genome to identify SNPs and insertion‐deletions. Candidate linkage intervals were determined by calculating the ΔSNP‐index across the genome with a 95% confidence threshold.

### Map‐based cloning of *SDEF*

Initial mapping of *qSD7‐1* was conducted with an F_2_ population of 200 plants derived from a NIL‐K × HJX74 cross. Genetic effects on seed dormancy were analyzed via SAS as described by Pan et al. (2021). Subsequently, 3580 F_3_ individuals were screened for recombinants to fine map *qSD7‐1*. Homozygous recombinants were then used for substitution mapping across various cropping seasons to narrow down the target region. Genomic DNA fragments of candidate genes in NIL‐K and HJX74 were sequenced, and their expression patterns in mature seeds were examined. Gene annotation was performed by MSU Rice Genome Annotation Project Release 7 (http://rice.uga.edu/). Primers for markers developed based on sequence polymorphisms are listed in Table S4.

### Vector construction and plant transformation

For CRISPR/Cas9 knockout, *SDEF* target sites were designed using CRISPRGE (https://skl.scau.edu.cn) and cloned into intermediate vector SK‐gRNA, then subcloned into pC1300‐Cas9 by Golden Gate cloning to generate pC1300‐Cas9‐SDEF^NK^ and pC1300‐Cas9‐SDEF^NJ^. For overexpression, the full‐length *SDEF* CDS from NIL‐K was cloned into pCAMBIA1300 (KpnI/BamHI) under the *OsActin1* promoter, generating pCAMBIA1300‐Actin‐SDEF. Validated constructs were transformed into NIL‐K, NIL‐J, and HJX74 via *Agrobacterium tumefaciens* strain EHA105. Primers for vector construction are listed in Table S4.

### RNA extraction and qRT‐PCR analysis

Total RNA was extracted from various rice tissues and organs using the RaPure Plant RNA Kit (Magen, Guangzhou, China) and then reverse‐transcribed into cDNA using the *Evo M*‐*MLV* RT Kit with gDNA Clean (Accurate Biology, Changsha, China). qRT‐PCR was performed on a CFX 96 instrument (Bio‐Rad, Hercules, CA, USA) using SYBR Green *Pro Taq* HS Premix (Accurate Biology). *OsActin1* (*LOC_Os03g50885*) served as the internal control. Data were analyzed using the 2^−ΔΔCT^ method based on three biological and three technical replicates. Primers for qRT‐PCR are listed in Table S4.

### GUS staining and histological analysis

Tissues from *pSDEF::GUS* transgenic plants were harvested for GUS staining analysis. Dehulled seeds were incubated in GUS staining solution either whole or longitudinally bisected along the embryo. Samples were incubated at 37°C for 24 h, rinsed with distilled water, and destained in 70% ethanol. Images were captured using a ZEISS Stemi 508 stereomicroscope. For histological analysis, stained leaves and roots were fixed in FAA for 48 h, dehydrated through a graded ethanol series, and embedded in resin. Cross‐sections (2–4 μm) were prepared using a Leica RM2265 microtome and examined under a Motic BA310 light microscope.

### Subcellular localization

For subcellular localization, the *SDEF* CDS without a stop codon was amplified from NIL‐K and fused to the N‐terminal of green fluorescent protein (GFP) driven by the 35S promoter. The SDEF‐GFP construct and a nuclear marker were co‐transformed into rice protoplasts via electroporation, with an empty vector serving as the negative control. GFP fluorescence signals were visualized using an Olympus FV1000 confocal microscope.

### Yeast two‐hybrid assays

The CDSs of full‐length and truncated *SDEF*, as well as *Cullin*, were cloned into pGBKT7 to generate bait vectors, while *OSK1* and *Cullin* were cloned into pGADT7 as prey vectors. The constructs were co‐transformed into yeast strain AH109. Transformants were grown on SD/‐Leu/‐Trp medium at 30°C for 2 days, then screened on SD/‐Trp/‐His/‐Leu/‐Ade medium for 2–4 days. All procedures followed the Yeast Protocols Handbook (Clontech). The primers for Y2H are listed in Table S4.

### In vitro pull‐down assay

Recombinant His‐SDEF and MBP‐OSK1/MBP (negative control) were purified using Ni‐NTA Beads 6FF (Smart Lifesciences, Changzhou, China) and Amylose Resin (New England Biolabs, Ipswich, MA, USA), respectively. Purified His‐SDEF was incubated with MBP‐OSK1‐ or MBP‐bound resin at 4°C for 3 h. After five washes with TBS buffer containing 0.1% Tween‐20, the bound proteins were eluted and analyzed via Western blotting using anti‐His and anti‐MBP antibodies (Abmart, Shanghai, China 1:4000). Primers used are listed in Table S4.

### Bimolecular fluorescence complementary assays

Full‐length and truncated CDSs of *SDEF* were cloned into p2YC to fuse with the C‐terminal of yellow fluorescent protein (YFP), whereas that of *OSK1* was cloned into p2YN for N‐terminal YFP fusion. These constructs were transformed into *Agrobacterium tumefaciens* GV3101. After overnight growth, the paired constructs were combined in infiltration buffer (10 mM MgCl_2_, 10 mM MES, 0.1 mM acetosyringone, pH 5.6) and co‐infiltrated into *Nicotiana benthamiana* leaves using a needleless syringe. YFP fluorescence was imaged 36–48 h post‐infiltration using confocal microscopy (Zeiss LSM 7810 DUO or LSM 7 Live). The primers for BiFC are listed in Table S4.

### RNA‐seq analysis

Total RNA was extracted from 24‐h imbibed seeds of HJX74 and NIL‐J with three biological replicates using TRIzol reagent (Invitrogen, Carlsbad, CA, USA). The cDNA library was constructed and sequenced at Gene Denovo Biotechnology Co. (Guangzhou, China) on an Illumina HiSeq 2000 platform. Clean reads were mapped to the 9311 reference genome using TopHat. Gene expression was quantified as fragments per kilobase of exon per million reads mapped (FPKM) using RSEM. Differentially expressed genes (DEGs) were identified using DESeq2 (Love et al., 2014), applying a false discovery rate (FDR) <0.05 and a fold change ≥1.5.

### Measurement of ABA and IAA

Seeds of HJX74 and NIL‐K were collected at 10, 20, and 30 DAP and stored at −80°C. Endogenous ABA and IAA were extracted and quantified by Shanghai Luming Biotechnology (Shanghai, China) using 50 mg of freeze‐dried samples. The analysis was performed on a UPLC‐ESI‐MS/MS system (ACQUITY UPLC, Waters; API5500 MS, AB SCIEX), and data were processed using the Analyst 1.6.2 software. Three biological replicates were performed.

### Phylogenetic analysis

Protein sequence of SDEF and its homologs from GenBank were aligned using ClustalW, and a neighbor‐joining phylogenetic tree with 1000 bootstrap replicates was constructed in MEGA11 (Tamura et al., 2021). A parallel analysis was performed for *SDEF* DNA haplotypes.

### Haplotype and nucleotide diversity analysis

SNPs from the 1‐kb promoter and CDS of *SDEF* were obtained from the 3K Rice Database (http://www.rmbreeding.cn/Index/) (Wang et al., 2018), retaining variants with a minor allele frequency ≥0.01. Haplotypes represented by at least 30 accessions were analyzed. Nucleotide diversity of *SDEF* and its flanking regions (±20 kb) was calculated using ECOGEMS (https://venyao.xyz/ECOGEMS/) based on 2058 rice accessions (446 wild and 1612 cultivated) (Yao et al., 2019).

### Statistical methods

Pairwise and multiple‐group comparisons were performed using two‐tailed Student's *t*‐tests in Excel 2019 and one‐way ANOVA with Duncan's multiple‐range test in SPSS 23, respectively.

## ACCESSION NUMBERS

The sequence data used in this study are available in the EnsemblPlants database (https://plants.ensembl.org/index.html) and the MSU Rice Genome Annotation Project database (http://rice.plantbiology.msu.edu) under the following accession numbers: *SDEF*, *BGIOSGA025222*, *LOC_Os07g07520*; *OSK1*, *BGIOSGA035212*, *LOC_Os11g26910*; *Cullin*, *BGIOSGA001633*, *LOC_Os01g27150*; *OsPYL9*, *BGIOSGA023096*, *LOC_Os06g36670*; *OsPP2C67*, *BGIOSGA030014*, *LOC_Os09g14540*; *OsSAPK1*, *BGIOSGA010605*, *LOC_Os03g27280*; *OsbZIP10*, *BGIOSGA004837*, *LOC_Os01g64000*; *OsVP1*, *BGIOSGA000276*, *LOC_Os01g68370*; *OsMFT2*, *BGIOSGA007632*, *LOC_Os01g02120*; *Sdr4*, *BGIOSGA026033*, *LOC_Os07g39700*; *OsDOG1L‐2*, *BGIOSGA017575*, *LOC_Os05g48650*; *OsDOG1L‐1*, *BGIOSGA004490* (no MSU ID available); *OsLEA14*, *BGIOSGA000945*, *LOC_Os01g50910*; *OsGA20ox1*, *BGIOSGA013985*, *LOC_Os03g63970*; *OsAMY1A*, *BGIOSGA005550*, *LOC_Os02g52710*; *OsAMY3D*, *BGIOSGA028835*, *LOC_Os08g36910*; *OsAMY3E*, *BGIOSGA028834*, *LOC_Os08g36900*; *OsAGLU1*, *BGIOSGA023458*, *LOC_Os06g46340*; *OsXTH2*, *BGIOSGA035393*, *LOC_Os11g33270*; *OsGH3.13*, *BGIOSGA033883*, *LOC_Os11g32520*; *OsUGT75K1*, *BGIOSGA002259*, *LOC_Os01g08440*; and *OsETR4*, *BGIOSGA024506*, *LOC_Os07g15540*.

## AUTHOR CONTRIBUTIONS

YZ conceived and designed the research. YW, GZ, CL, YZ, WL, RH, HK, XC, and ML performed most of the experiments. CL and RZ conducted the initial fine mapping. HC and BT provided technical support. HL and ZX assisted with the field experiments. YZ performed the majority of data analysis and visualization, assisted by YW. YZ drafted and revised the manuscript. All authors read and approved the final manuscript.

## CONFLICT OF INTEREST

The authors declare no conflicts of interest.

## Supporting information


**Figure S1.** Initial mapping of *qSD7‐1*. (a) Frequency distribution of *qSD7‐1* in a 200 F_2_ segregating population derived from HJX74 and NIL‐K. The donor parent of NIL‐K is Khazar. ‘aa’ represents the HJX74 allele of *qSD7‐1*, and ‘AA’ represents the Khazar allele of *qSD7‐1*. SD was evaluated after 7 days imbibition. The genotypes of individual plants were determined by the InDel Ui46 marker. The additive effects (*a*), dominance effects (*d*), and proportion of the variance explained by the QTL (*R* ^2^) were calculated by SAS program. A negative *a* or *d* value indicates that the ‘A’ allele decreased germination. **** *P* < 0.0001, Student's *t*‐test. (b) The genetic linkage map of *qSD7‐1* locus on Chromosome 7 based on 200 plants. Markers and genetic distances (cM) are indicated on the right and on the left of the chromosome, respectively. Chr, chromosome.
**Figure S2.** DNA sequence alignment of *SDEF* between HJX74 and NIL‐K. UTRs were shaded in green, exons in black, introns in gray, and the single‐nucleotide substitution in red. Nip, Nipponbare.
**Figure S3.** Temporal and spatial expression profiles of candidate genes in *qSD7‐1*. (a) A heat map of the relative expression of nine candidate genes. All expression data were prepared by the web tool RiceXPro (http://ricexpro.dna.affrc.go.jp/). The gray boxes mean no expression data. The heat map was made by TBtools (v2.016). (b) The relative expression of nine candidate genes by qRT‐PCR. *OsUBC* (*LOC_Os02g42314*) was used as a reference gene. Data are means ± SD (n = three biological replicates). *P < 0.05, **P < 0.01, Student's *t*‐test. ns, not significant. DAP, days after pollination.
**Figure S4.** Comparison of the major agronomic traits of HJX74, NIL‐K, and NIL‐J. (a–g) Comparison of plant height (a), effective panicle number (b), grain number per panicle (c), grain length (d), grain width (e), 1000‐grains weight (f), and yield per plant (g) between HJX74, NIL‐K and NIL‐J. Data are means ± SD. *n* = 58 (a), 15 (b), 10 (c), 5 (d), 5 (e), 15 (f), 10 (g) plants. *P < 0.05, **P < 0.01, ***P < 0.001, ****P < 0.0001. ns, not significant. Student's *t*‐test.
**Figure S5.** F‐box domain analysis of SDEF. (a) Schematic structure of the SDEF protein. The red rectangle shows the F‐box like domain (FBLD). (b) F‐box domain alignment of SDEF and its homologs. Identical amino acids are shaded in black and conserved changes in red. Homologs are from *Panicum virgatum* (XP_039784084.1), *Setaria italica* (XP_004982956.1), *Triticum aestivum* (XP_044395551.1), *Hordeum vulgare* (XP_044970079.1), *Aegilops tauschii* (XP_020165376.1), *Brachypodium distachyon* (XP_003574011.1), *Zea mays* (ACG42305.1), and *Sorghum bicolor* (XP_002467126.1).
**Figure S6.** Protein sequence multiple alignment of SDEF with its homologs. Identical amino acids are shaded in black and conserved changes in gray. The conserved F‐box‐like domain is underlined. The tryptophan (Trp, W) at position 153, a natural variation between NIL‐K and HJX74, is indicated by an asterisk. All sequences were obtained from the NCBI GenBank database. Accession numbers and species are indicated as follows: XP_039784084.1 (*Panicum virgatum*), XP_004982956.1 (*Setaria italica*), XP_044395551.1 (*Triticum aestivum*), XP_044970079.1 (*Hordeum vulgare*), XP_020165376.1 (*Aegilops tauschii*), XP_003574011.1 (*Brachypodium distachyon*), ACG42305.1 (*Zea mays*), and XP_002467126.1 (*Sorghum bicolor*).
**Figure S7.** Phylogenetic tree of SDEF and other F‐box proteins in flowering plants. Bootstrap values (1000 replicates) are shown at nodes. SDEF is highlighted in red.
**Figure S8.** *pSDEF::GUS* staining in young root (a, b), reproductive stem (c) and young leaf (d, e). Cross‐sections for root (b) and leaf (e) are shown. Scale bars, 1 mm (a, d), 2 mm (c) and 50 μm (b, e).
**Figure S9.** Y2H assays showing the interactions of Cullin with SDEF and OSK1. Yeast transformants were grown on control SD‐WL (‐Trp/‐Leu) or selective SD‐WLHA (‐Trp/‐Leu/‐His/‐Ade) media.
**Figure S10.** Comparison of ABA (a) and IAA (b) content in the developing seeds between HJX74 and NIL‐K. FW, fresh weight. Data are means ± SD (*n* = three biological replicates). *P < 0.05, **P < 0.01, ***P < 0.001, ****P < 0.0001. ns, not significant. Student's *t*‐test.
**Figure S11.** Germination percentages of freshly harvested panicles in different rice subpopulations. Data are means ± SE after 7 days imbibition. *n* = 4 (IND I), 12 (IND II), 25 (IND III), 9 (IND INT), 3 (AUS), 3 (INT), 3 (TEJ), and 2 (TRJ) accessions. IND I, *indica* I; IND II, *indica* II; IND III, *indica* III; IND INT, *indica* intermediate; AUS, *aus*; INT, intermediate; TEJ, temperate *japonica*; TRJ, tropical *japonica*.
**Figure S12.** Subpopulation‐matched comparison of seed dormancy among Hap2, Hap3, and Hap5. Germination rates of freshly harvested panicles were compared within the same IND III and IND INT subpopulations. Data are means ± SE after 7 days imbibition. *n* = 13 (Hap2), 6 (Hap3), and 13 (Hap5) accessions. Each accession contains 10 independent plants. Different letters denote significant differences (*P* < 0.05, Duncan's multiple‐range test).
**Figure S13.** Phenotypic comparison of seed dormancy between Hap1 (457‐T) and the rare Hap (457‐C) accessions. Data are means ± SE after 7 days imbibition. *n* = 20 (Hap1, 457‐T) and 10 (rare Hap, 457‐C) accessions. Each accession contains 10 independent plants. Student's *t*‐test. ns, not significant.

**Table S1.** Predicted genes at the *qSD7‐1* locus.
**Table S2.** DEGs between HJX74 and NIL‐J in 24 h‐imbibed seeds in rice.
**Table S3.** SNPs information and haplotypes of *SDEF* for 2420 3K rice accessions.
**Table S4.** Primers used in this study.

## Data Availability

The raw RNA‐seq data have been deposited in the Genome Sequence Archive (GSA) at the National Genomics Data Center under accession number CRA047007 (https://ngdc.cncb.ac.cn/gsa). The data that support the findings of this study are included within the article and its Supporting Information files.
