# Supplementary material for: Natural variation in the evolutionarily conserved F‐box gene SDEF promotes seed dormancy in rice
Source: Plant J. 2026 Oct 30;128(3):e71203. doi: 10.1111/tpj.71203 (PMC13720509; doi:10.1111/tpj.71203)
Supplement: Supplementary file 1 [file TPJ-128-0-s001.docx]

**Fig. S1** **Initial mapping of *qSD7-1*. a**, Frequency distribution of *qSD7-1* in a 200 F_2_ segregating population derived from HJX74 and NIL-K. The donor parent of NIL-K is Khazar. "aa" represents the HJX74 allele of *qSD7-1*, and "AA" represents the Khazar allele of *qSD7-1*. SD was evaluated after 7 d imbibition. The genotypes of individual plants were determined by the InDel Ui46 marker. The additive effects (*a*), dominance effects (*d*), and proportion of the variance explained by the QTL (*R*^2^) were calculated by SAS program. A negative *a* or *d* value indicates that the "A" allele decreased germination. **** *P* < 0.0001, Student’s *t*-test. **b**, The genetic linkage map of *qSD7-1* locus on chromosome 7 based on 200 plants. Markers and genetic distances (cM) are indicated on the right and on the left of the chromosome, respectively. Chr, chromosome.

CDS_Nip	---------------------------------------------------------------------- 0

DNA_Nip	AGCATCGCATCGCCTCGCCCACATACGAAGGACCGTGACGAGGCGCGTTGACACGCACGCATGCGTGGGT 70

DNA_NIL-K	AGCATCGCATCGCCTCGCCCACATACGAAGGACCGTGACGAGGCGCGTTGACACGCACGCATGCGTGGGT 70

DNA_HJX74	AGCATCGCATCGCCTCGCCCACATACGAAGGACCGTGACGAGGCGCGTTGACACGCACGCATGCGTGGGT 70

CDS_Nip	---------------------------------------------------------------------- 0

DNA_Nip	GTCCACGTGGGCAGCATACGTGCGAGAGCTCCCCCGCCGCCGGATCGCCGGAGCCCGCCTCATCCGTCCG 140

DNA_NIL-K	GTCCACGTGGGCAGCATACGTGCGAGAGCTCCCCCGCCGCCGGATCGCCGGAGCCCGCCTCATCCGTCCG 140

DNA_HJX74	GTCCACGTGGGCAGCATACGTGCGAGAGCTCCCCCGCCGCCGGATCGCCGGAGCCCGCCTCATCCGTCCG 140

CDS_Nip	---------------------------------------------------------------------- 0

DNA_Nip	TCCGTTCATCCCGTCCGGCGCCGCGGCTCAATAAATGCGGGCCCCAACGAGCTAAATCTTCCCAAAAGGG 210

DNA_NIL-K	TCCGTTCATCCCGTCCGGCGCCGCGGCTCAATAAATGCGGGCCCCAACGAGCTAAATCTTCCCAAAAGGG 210

DNA_HJX74	TCCGTTCATCCCGTCCGGCGCCGCGGCTCAATAAATGCGGGCCCCAACGAGCTAAATCTTCCCAAAAGGG 210

CDS_Nip	---------------------------------------------------------------------- 0

DNA_Nip	AGCTGAGTAGATAGATACTCCTCACTCCTCCACTCCGCTCTGCTGCTGCTGCTCTCGTCGTGGTGGTGGG 280

DNA_NIL-K	AGCTGAGTAGATAGATACTCCTCACTCCTCCACTCCGCTCTGCTGCTGCTGCTCTCGTCGTGGTGGTGGG 280

DNA_HJX74	AGCTGAGTAGATAGATACTCCTCACTCCTCCACTCCGCTCTGCTGCTGCTGCTCTCGTCGTGGTGGTGGG 280

CDS_Nip	-ATGGCGGAGGAGCGGAGGAGGCGGAAGCGGGAGGAGGAGGAGGAGGCGGAGGAGTGGGAGAGGCGGAAG 69

DNA_Nip	AATGGCGGAGGAGCGGAGGAGGCGGAAGCGGGAGGAGGAGGAGGAGGCGGAGGAGTGGGAGAGGCGGAAG 350

DNA_NIL-K	AATGGCGGAGGAGCGGAGGAGGCGGAAGCGGGAGGAGGAGGAGGAGGCGGAGGAGTGGGAGAGGCGGAAG 350

DNA_HJX74	AATGGCGGAGGAGCGGAGGAGGCGGAAGCGGGAGGAGGAGGAGGAGGCGGAGGAGTGGGAGAGGCGGAAG 350

CDS_Nip	AGGGGGAGGAGGAAGAGGAGGAAGAGGAGGAGGAGGCGGGGAGGGGAGGAGGACCCCGTGGATGTGCTCG 139

DNA_Nip	AGGGGGAGGAGGAAGAGGAGGAAGAGGAGGAGGAGGCGGGGAGGGGAGGAGGACCCCGTGGATGTGCTCG 420

DNA_NIL-K	AGGGGGAGGAGGAAGAGGAGGAAGAGGAGGAGGAGGCGGGGAGGGGAGGAGGACCCCGTGGATGTGCTCG 420

DNA_HJX74	AGGGGGAGGAGGAAGAGGAGGAAGAGGAGGAGGAGGCGGGGAGGGGAGGAGGACCCCGTGGATGTGCTCG 420

CDS_Nip	GGGAGGAGGTGATGGGGCGGGTGATGGAGCTCCTGGACGCACGCAGCGTGGCACGATGCACCGCGGTGTC 209

DNA_Nip	GGGAGGAGGTGATGGGGCGGGTGATGGAGCTCCTGGACGCACGCAGCGTGGCACGATGCACCGCGGTGTC 490

DNA_NIL-K	GGGAGGAGGTGATGGGGCGGGTGATGGAGCTCCTGGACGCACGCAGCGTGGCACGATGCACCGCGGTGTC 490

DNA_HJX74	GGGAGGAGGTGATGGGGCGGGTGATGGAGCTCCTGGACGCACGCAGCGTGGCACGATGCACCGCGGTGTC 490

CDS_Nip	CCGCGCCTGGCGCGGGGTCGCCGCCGACGACCGCCTCTGGGCACCCAAG--------------------- 258

DNA_Nip	CCGCGCCTGGCGCGGGGTCGCCGCCGACGACCGCCTCTGGGCACCCAAGGTCCCCCCTCTCTCTCTCTTT 560

DNA_NIL-K	CCGCGCCTGGCGCGGGGTCGCCGCCGACGACCGCCTCTGGGCACCCAAGGTCCCCCCTCTCTCTCTCTTT 560

DNA_HJX74	CCGCGCCTGGCGCGGGGTCGCCGCCGACGACCGCCTCTGGGCACCCAAGGTCCCCCCTCTCTCTCTCTTT 560

CDS_Nip	---------------------------------------------------------------------- 258

DNA_Nip	CTCTCTCGTTACAGCCTGCACGGCGCCTGTTCGACGAATTGCCGGCGAGGGGCCTCCGATTCCCCTCACG 630

DNA_NIL-K	CTCTCTCGTTACAGCCTGCACGGCGCCTGTTCGACGAATTGCCGGCGAGGGGCCTCCGATTCCCCTCACG 630

DNA_HJX74	CTCTCTCGTTACAGCCTGCACGGCGCCTGTTCGACGAATTGCCGGCGAGGGGCCTCCGATTCCCCTCACG 630

CDS_Nip	---------------------------------------------------------------------- 258

DNA_Nip	GGCGGTGGCTACTCTCGTGTCTTCTGTTAGGATCGAGCTTATCGTATTCGGGAGTGGTGTTGATTTGGAG 700

DNA_NIL-K	GGCGGTGGCTACTCTCGTGTCTTCTGTTAGGATCGAGCTTATCGTATTCGGGAGTGGTGTTGATTTGGAG 700

DNA_HJX74	GGCGGTGGCTACTCTCGTGTCTTCTGTTAGGATCGAGCTTATCGTATTCGGGAGTGGTGTTGATTTGGAG 700

CDS_Nip	---------------------------------------------------------------------- 258

DNA_Nip	CTTTTATAGAATTCTCGTTTATTGATTATCAAATTATGGTTGCCAAAAAGAGAAGAGCGCACATTCGGCT 770

DNA_NIL-K	CTTTTATAGAATTCTCGTTTATTGATTATCAAATTATGGTTGCCAAAAAGAGAAGAGCGCACATTCGGCT 770

DNA_HJX74	CTTTTATAGAATTCTCGTTTATTGATTATCAAATTATGGTTGCCAAAAAGAGAAGAGCGCACATTCGGCT 770

CDS_Nip	---------------------------------------------------------------------- 258

DNA_Nip	AGTGTGTTAGTACTGTTTTTGGTACAAAGGTCGTTATATATATAAACCCAAGAGGCTCGTTGTTGATTGC 840

DNA_NIL-K	AGTGTGTTAGTACTGTTTTTGGTACAAAGGTCGTTATATATATAAACCCAAGAGGCTCGTTGTTGATTGC 840

DNA_HJX74	AGTGTGTTAGTACTGTTTTTGGTACAAAGGTCGTTATATATATAAACCCAAGAGGCTCGTTGTTGATTGC 840

CDS_Nip	---------------------------------------------------------------------- 258

DNA_Nip	TCCTGTGCCCGTAGCATGGCGAGATAAGACAGAGAGAGTACGGCGTGTGACAGACGTACTGTACAAACTT 910

DNA_NIL-K	TCCTGTGCCCGTAGCATGGCGAGATAAGACAGAGAGAGTACGGCGTGTGACAGACGTACTGTACAAACTT 910

DNA_HJX74	TCCTGTGCCCGTAGCATGGCGAGATAAGACAGAGAGAGTACGGCGTGTGACAGACGTACTGTACAAACTT 910

CDS_Nip	---------------------------------------------------------------------- 258

DNA_Nip	GTTGTGACATTGACATGGTAGGAAAACAAGTACCTTTCCCGACAGGGATAACGATCTTTCTTCTATTCCC 980

DNA_NIL-K	GTTGTGACATTGACATGGTAGGAAAACAAGTACCTTTCCCGACAGGGATAACGATCTTTCTTCTATTCCC 980

DNA_HJX74	GTTGTGACATTGACATGGTAGGAAAACAAGTACCTTTCCCGACAGGGATAACGATCTTTCTTCTATTCCC 980

CDS_Nip	---------------------------------------------------------------------- 258

DNA_Nip	CCCAAGAGAGACAAAATTACGACAATAAGAAATGATAATCACACTGAATATTTTCTATCATTTTGCTCTG 1050

DNA_NIL-K	CCCAAGAGAGACAAAATTACGACAATAAGAAATGATAATCACACTGAATATTTTCTATCATTTTGCTCTG 1050

DNA_HJX74	CCCAAGAGAGACAAAATTACGACAATAAGAAATGATAATCACACTGAATATTTTCTATCATTTTGCTCTG 1050

CDS_Nip	---------------------------------------------------------------------- 258

DNA_Nip	TCTATTCCTAAATTTTGAGTTGTTGTTCAATTTGATTTTCAACCAATCTTAGAATATTTCATATTTTAAC 1120

DNA_NIL-K	TCTATTCCTAAATTTTGAGTTGTTGTTCAATTTGATTTTCAACCAATCTTAGAATATTTCATATTTTAAC 1120

DNA_HJX74	TCTATTCCTAAATTTTGAGTTGTTGTTCAATTTGATTTTCAACCAATCTTAGAATATTTCATATTTTAAC 1120

CDS_Nip	---------------------------------------------------------------------- 258

DNA_Nip	AATTGACGGATCTTTGTATAGAGATGATTGTGATTTTGTAAAAAATGTTCATTTTTAGTCTGACTTACAT 1190

DNA_NIL-K	AATTGACGGATCTTTGTATAGAGATGATTGTGATTTTGTAAAAAATGTTCATTTTTAGTCTGACTTACAT 1190

DNA_HJX74	AATTGACGGATCTTTGTATAGAGATGATTGTGATTTTGTAAAAAATGTTCATTTTTAGTCTGACTTACAT 1190

CDS_Nip	---------------------------------------------------------------------- 258

DNA_Nip	TGAAGATCCTGAATTTCCATAATATCAATATATATAAAAGTCTGTGTATTTTAGATAGTCCTTTTTTTTT 1260

DNA_NIL-K	TGAAGATCCTGAATTTCCATAATATCAATATATATAAAAGTCTGTGTATTTTAGATAGTCCTTTTTTTTT 1260

DNA_HJX74	TGAAGATCCTGAATTTCCATAATATCAATATATATAAAAGTCTGTGTATTTTAGATAGTCCTTTTTTTTT 1260

CDS_Nip	---------------------------------------------------------------------- 258

DNA_Nip	GTAATTTTCTTGGATTTTATTACTCGTGCCGATGGACACTCATGTCAAAGGTAGGTTGGGTGCCTCATGT 1330

DNA_NIL-K	GTAATTTTCTTGGATTTTATTACTCGTGCCGATGGACACTCATGTCAAAGGTAGGTTGGGTGCCTCATGT 1330

DNA_HJX74	GTAATTTTCTTGGATTTTATTACTCGTGCCGATGGACACTCATGTCAAAGGTAGGTTGGGTGCCTCATGT 1330

CDS_Nip	---------------------------------------------------------------------- 258

DNA_Nip	AGTCATGTTTATGTCACCTTGAGCAGAAAGCCAGAAACCATGAGAAAGAGGAAACAAAACTTAACTAAAT 1400

DNA_NIL-K	AGTCATGTTTATGTCACCTTGAGCAGAAAGCCAGAAACCATGAGAAAGAGGAAACAAAACTTAACTAAAT 1400

DNA_HJX74	AGTCATGTTTATGTCACCTTGAGCAGAAAGCCAGAAACCATGAGAAAGAGGAAACAAAACTTAACTAAAT 1400

CDS_Nip	---------------------------------------------------------------------- 258

DNA_Nip	TCTCATGAACTAAAGAAAGTACAACAACAAAAATAGAAGTATTATAGGGATTGATATGAAAAATTAAAAC 1470

DNA_NIL-K	TCTCATGAACTAAAGAAAGTACAACAACAAAAATAGAAGTATTATAGGGATTGATATGAAAAATTAAAAC 1470

DNA_HJX74	TCTCATGAACTAAAGAAAGTACAACAACAAAAATAGAAGTATTATAGGGATTGATATGAAAAATTAAAAC 1470

CDS_Nip	---------------------------------------------------------------------- 258

DNA_Nip	CATCATTAAAGTCAATTTGCTTCTGTCCGTCTTAGAAACTAGGGATTGCTTTGCACTGCAAATGTTCATA 1540

DNA_NIL-K	CATCATTAAAGTCAATTTGCTTCTGTCCGTCTTAGAAACTAGGGATTGCTTTGCACTGCAAATGTTCATA 1540

DNA_HJX74	CATCATTAAAGTCAATTTGCTTCTGTCCGTCTTAGAAACTAGGGATTGCTTTGCACTGCAAATGTTCATA 1540

CDS_Nip	---------------------------------------------------------------------- 258

DNA_Nip	GAGGGAATATCTATAAAACATAGAACACTCTTTTTATAAGCAATAATCTATAATCTCTGTCTCAAAATAG 1610

DNA_NIL-K	GAGGGAATATCTATAAAACATAGAACACTCTTTTTATAAGCAATAATCTATAATCTCTGTCTCAAAATAG 1610

DNA_HJX74	GAGGGAATATCTATAAAACATAGAACACTCTTTTTATAAGCAATAATCTATAATCTCTGTCTCAAAATAG 1610

CDS_Nip	---------------------------------------------------------------------- 258

DNA_Nip	ATTGCTATAATTTAAGACGGAGGAAATAACGTGATTACTATGCGGCTGTCGACATGTACAGAGTGCACTG 1680

DNA_NIL-K	ATTGCTATAATTTAAGACGGAGGAAATAACGTGATTACTATGCGGCTGTCGACATGTACAGAGTGCACTG 1680

DNA_HJX74	ATTGCTATAATTTAAGACGGAGGAAATAACGTGATTACTATGCGGCTGTCGACATGTACAGAGTGCACTG 1680

CDS_Nip	---------------------------------------------------------------------- 258

DNA_Nip	ATACACACACCCATCCATCCAACCCGTTTCGTCTTTTGTGGCTCCAGACTTCCGAGCTAAGCATCGCCCA 1750

DNA_NIL-K	ATACACACACCCATCCATCCAACCCGTTTCGTCTTTTGTGGCTCCAGACTTCCGAGCTAAGCATCGCCCA 1750

DNA_HJX74	ATACACACACCCATCCATCCAACCCGTTTCGTCTTTTGTGGCTCCAGACTTCCGAGCTAAGCATCGCCCA 1750

CDS_Nip	---------------------------------------------------------------------- 258

DNA_Nip	TAAAAAGCAACCCAACCCACACATGCATGTGTCCACGTGGGCACCCTGGCACGCTAGCTGCCACGTAGGA 1820

DNA_NIL-K	TAAAAAGCAACCCAACCCACACATGCATGTGTCCACGTGGGCACCCTGGCACGCTAGCTGCCACGTAGGA 1820

DNA_HJX74	TAAAAAGCAACCCAACCCACACATGCATGTGTCCACGTGGGCACCCTGGCACGCTAGCTGCCACGTAGGA 1820

CDS_Nip	---------------------------------------------------------------------- 258

DNA_Nip	ACACCCCTCTTGCCGCCACCCGCTCCTCCGCCACTGGATCGCCAGAGCTCCCCCTCATCCTTCCATCCGT 1890

DNA_NIL-K	ACACCCCTCTTGCCGCCACCCGCTCCTCCGCCACTGGATCGCCAGAGCTCCCCCTCATCCTTCCATCCGT 1890

DNA_HJX74	ACACCCCTCTTGCCGCCACCCGCTCCTCCGCCACTGGATCGCCAGAGCTCCCCCTCATCCTTCCATCCGT 1890

CDS_Nip	---------------------------------------------------------------------- 258

DNA_Nip	CTGACACCCTGCGCCTCTATAAATGCGCGCCCCAACGACCTTCCCCAAAAGGCAAGCGATCTGCGCCACG 1960

DNA_NIL-K	CTGACACCCTGCGCCTCTATAAATGCGCGCCCCAACGACCTTCCCCAAAAGGCAAGCGATCTGCGCCACG 1960

DNA_HJX74	CTGACACCCTGCGCCTCTATAAATGCGCGCCCCAACGACCTTCCCCAAAAGGCAAGCGATCTGCGCCACG 1960

CDS_Nip	---------------------------------------------------------------------- 258

DNA_Nip	GCAACGGCCCCACCTAGCTGAGTAGATCACTCACTCACTCCTCCCCTCCTCTCCTCTCCACTCTGCTCTG 2030

DNA_NIL-K	GCAACGGCCCCACCTAGCTGAGTAGATCACTCACTCACTCCTCCCCTCCTCTCCTCTCCACTCTGCTCTG 2030

DNA_HJX74	GCAACGGCCCCACCTAGCTGAGTAGATCACTCACTCACTCCTCCCCTCCTCTCCTCTCCACTCTGCTCTG 2030

CDS_Nip	---------------------------------------------------------------------- 258

DNA_Nip	CTCTGCTGCTCGGCTCTGCTTGTATTCCTCACACGAGCGTTTAATTTAGCATTGACTGCTCTCATGCCCT 2100

DNA_NIL-K	CTCTGCTGCTCGGCTCTGCTTGTATTCCTCACACGAGCGTTTAATTTAGCATTGACTGCTCTCATGCCCT 2100

DNA_HJX74	CTCTGCTGCTCGGCTCTGCTTGTATTCCTCACACGAGCGTTTAATTTAGCATTGACTGCTCTCATGCCCT 2100

CDS_Nip	---------------------------------------------------------------------- 258

DNA_Nip	CTGTTAGGATCAGGATCATATTGGAGGGGATGTTGATTTCAAGCAAGCATTCGGCTAGTACAGTTAACAG 2170

DNA_NIL-K	CTGTTAGGATCAGGATCATATTGGAGGGGATGTTGATTTCAAGCAAGCATTCGGCTAGTACAGTTAACAG 2170

DNA_HJX74	CTGTTAGGATCAGGATCATATTGGAGGGGATGTTGATTTCAAGCAAGCATTCGGCTAGTACAGTTAACAG 2170

CDS_Nip	---------------------------------------------------------------------- 258

DNA_Nip	TCACACAGTTTGGTACCAACTACCAAGGTTATTATCTAAATCCCAAAAGGGTAATTGTTGACTCGTGGAA 2240

DNA_NIL-K	TCACACAGTTTGGTACCAACTACCAAGGTTATTATCTAAATCCCAAAAGGGTAATTGTTGACTCGTGGAA 2240

DNA_HJX74	TCACACAGTTTGGTACCAACTACCAAGGTTATTATCTAAATCCCAAAAGGGTAATTGTTGACTCGTGGAA 2240

CDS_Nip	---------------------------------------------------------------------- 258

DNA_Nip	GTGCGAGATAAGAAGGGATGTTCGGTGTGTGACAGACTCATGTGTACAAACTTTGTGTGACATTGACATG 2310

DNA_NIL-K	GTGCGAGATAAGAAGGGATGTTCGGTGTGTGACAGACTCATGTGTACAAACTTTGTGTGACATTGACATG 2310

DNA_HJX74	GTGCGAGATAAGAAGGGATGTTCGGTGTGTGACAGACTCATGTGTACAAACTTTGTGTGACATTGACATG 2310

CDS_Nip	---------------------------------------------------------------------- 258

DNA_Nip	GTGGGAAAAAAAACAATTATTCTATCAGACAGGGATAAAGTTGTTTCTTTGATTCCCCGAAAAGATACAA 2380

DNA_NIL-K	GTGGGAAAAAAAACAATTATTCTATCAGACAGGGATAAAGTTGTTTCTTTGATTCCCCGAAAAGATACAA 2380

DNA_HJX74	GTGGGAAAAAAAACAATTATTCTATCAGACAGGGATAAAGTTGTTTCTTTGATTCCCCGAAAAGATACAA 2380

CDS_Nip	---------------------------------------------------------------------- 258

DNA_Nip	AATCGCAGCAATGATAAACGAGACGCTTTGAAATATTTTCTATTAGATAGAACTTTTCCAAGTGTAATTC 2450

DNA_NIL-K	AATCGCAGCAATGATAAACGAGACGCTTTGAAATATTTTCTATTAGATAGAACTTTTCCAAGTGTAATTC 2450

DNA_HJX74	AATCGCAGCAATGATAAACGAGACGCTTTGAAATATTTTCTATTAGATAGAACTTTTCCAAGTGTAATTC 2450

CDS_Nip	---------------------------------------------------------------------- 258

DNA_Nip	TTGTTTTTCTTTTAAGTATTGATCTTTTTTATTTTTCGATTAATCTTGGCTGTCAATGGTATCATAGTAT 2520

DNA_NIL-K	TTGTTTTTCTTTTAAGTATTGATCTTTTTTATTTTTCGATTAATCTTGGCTGTCAATGGTATCATAGTAT 2520

DNA_HJX74	TTGTTTTTCTTTTAAGTATTGATCTTTTTTATTTTTCGATTAATCTTGGCTGTCAATGGTATCATAGTAT 2520

CDS_Nip	---------------------------------------------------------------------- 258

DNA_Nip	TTTGTGCACATTTCATCTTTTAACAATTGACATCTCTAGGAGTATTTTGTGCAGTATATGGACAATCGTG 2590

DNA_NIL-K	TTTGTGCACATTTCATCTTTTAACAATTGACATCTCTAGGAGTATTTTGTGCAGTATATGGACAATCGTG 2590

DNA_HJX74	TTTGTGCACATTTCATCTTTTAACAATTGACATCTCTAGGAGTATTTTGTGCAGTATATGGACAATCGTG 2590

CDS_Nip	---------------------------------------------------------------------- 258

DNA_Nip	CATTTCTCTGAAAGTATTAATTTAGTCTTATCCATATTGATTATCCTGAATTTCCACGATATCAATTAAT 2660

DNA_NIL-K	CATTTCTCTGAAAGTATTAATTTAGTCTTATCCATATTGATTATCCTGAATTTCCACGATATCAATTAAT 2660

DNA_HJX74	CATTTCTCTGAAAGTATTAATTTAGTCTTATCCATATTGATTATCCTGAATTTCCACGATATCAATTAAT 2660

CDS_Nip	---------------------------------------------------------------------- 258

DNA_Nip	TTACATAAAAAATCATATATTTTATTTGTTTATGTATTGTCGTTTTTTTGTTTTCAATTTTCTAGGATTT 2730

DNA_NIL-K	TTACATAAAAAATCATATATTTTATTTGTTTATGTATTGTCGTTTTTTTGTTTTCAATTTTCTAGGATTT 2730

DNA_HJX74	TTACATAAAAAATCATATATTTTATTTGTTTATGTATTGTCGTTTTTTTGTTTTCAATTTTCTAGGATTT 2730

CDS_Nip	---------------------------------------------------------------------- 258

DNA_Nip	AAACACTCACAGCAATGAAATAAAAGTCGCTCATGACTAATGTCAAAGGTAGTTCAGGTGGCTCATATTT 2800

DNA_NIL-K	AAACACTCACAGCAATGAAATAAAAGTCGCTCATGACTAATGTCAAAGGTAGTTCAGGTGGCTCATATTT 2800

DNA_HJX74	AAACACTCACAGCAATGAAATAAAAGTCGCTCATGACTAATGTCAAAGGTAGTTCAGGTGGCTCATATTT 2800

CDS_Nip	---------------------------------------------------------------------- 258

DNA_Nip	TTGTTACCGTAAGCAGACCCATGACCATGAAACAGTGGAAAAGACTTAATTCAGCTCAAATTTAGCTTCA 2870

DNA_NIL-K	TTGTTACCGTAAGCAGACCCATGACCATGAAACAGTGGAAAAGACTTAATTCAGCTCAAATTTAGCTTCA 2870

DNA_HJX74	TTGTTACCGTAAGCAGACCCATGACCATGAAACAGTGGAAAAGACTTAATTCAGCTCAAATTTAGCTTCA 2870

CDS_Nip	---------------------------------------------------------------------- 258

DNA_Nip	GAATCTGAGTCTCCAATACTCCTATTTTGAAGTTACGAGTTCTTGCATTCATTTAACAAGGGAATGCAGA 2940

DNA_NIL-K	GAATCTGAGTCTCCAATACTCCTATTTTGAAGTTACGAGTTCTTGCATTCATTTAACAAGGGAATGCAGA 2940

DNA_HJX74	GAATCTGAGTCTCCAATACTCCTATTTTGAAGTTACGAGTTCTTGCATTCATTTAACAAGGGAATGCAGA 2940

CDS_Nip	---------------------------------------------------------------------- 258

DNA_Nip	TCCCAAATGTAAACTGAGAATTCTTCATTAAATATAGAGAGGGGAGAAGGGGTGCTATGGTTAATAATAC 3010

DNA_NIL-K	TCCCAAATGTAAACTGAGAATTCTTCATTAAATATAGAGAGGGGAGAAGGGGTGCTATGGTTAATAATAC 3010

DNA_HJX74	TCCCAAATGTAAACTGAGAATTCTTCATTAAATATAGAGAGGGGAGAAGGGGTGCTATGGTTAATAATAC 3010

CDS_Nip	----------------------------------------------------TGTGCTGAATTGATGGCG 276

DNA_Nip	TATTTCTTTGCTGACAGTCACAAGCAAATCTTGACCATTGTTTTATCTGCAGTGTGCTGAATTGATGGCG 3080

DNA_NIL-K	TATTTCTTTGCTGACAGTCACAAGCAAATCTTGACCATTGTTTTATCTGCAGTGTGCTGAATTGATGGCG 3080

DNA_HJX74	TATTTCTTTGCTGACAGTCACAAGCAAATCTTGACCATTGTTTTATCTGCAGTGTGCTGAATTGATGGCG 3080

CDS_Nip	GGAAAGGCACACATTCCTCGTTTGACTATGATACCCACTGCATCAAAATTGTCTACCTATTCAATGGCCA 346

DNA_Nip	GGAAAGGCACACATTCCTCGTTTGACTATGATACCCACTGCATCAAAATTGTCTACCTATTCAATGGCCA 3150

DNA_NIL-K	GGAAAGGCACACATTCCTCGTTTGACTATGATACCCACTGCATCAAAATTGTCTACCTATTCAATGGCCA 3150

DNA_HJX74	GGAAAGGCACACATTCCTCGTTTGACTATGATACCCACTGCATCAAAATTGTCTACCTATTCAATGGCCA 3150

CDS_Nip	TCGCGGATGGCAAACGG----------------------------------------------------- 363

DNA_Nip	TCGCGGATGGCAAACGGGTAAGTGCTAGCAAGGCAGCAATTTACTCTTGTGATATTTGTTAAAGATGCAA 3220

DNA_NIL-K	TCGCGGATGGCAAACGGGTAAGTGCTAGCAAGGCAGCAATTTACTCTTGTGATATTTGTTAAAGATGCAA 3220

DNA_HJX74	TCGCGGATGGCAAACGGGTAAGTGCTAGCAAGGCAGCAATTTACTCTTGTGATATTTGTTAAAGATGCAA 3220

CDS_Nip	---------------------------------------------------------------------- 363

DNA_Nip	TATATCAACAACATTGAGATGGTGATGCACTGTGTTGATGTGCTAGTGGTGCTTTAACCACAACTAAACG 3290

DNA_NIL-K	TATATCAACAACATTGAGATGGTGATGCACTGTGTTGATGTGCTAGTGGTGCTTTAACCACAACTAAACG 3290

DNA_HJX74	TATATCAACAACATTGAGATGGTGATGCACTGTGTTGATGTGCTAGTGGTGCTTTAACCACAACTAAACG 3290

CDS_Nip	---------------------------------------------------------------------- 363

DNA_Nip	AAAAGATAACCTCCGGACTTTTGGAGTAATTTGCCGCCATTTTCTTATCTTCTTTCCAGCAACCAAAACA 3360

DNA_NIL-K	AAAAGATAACCTCCGGACTTTTGGAGTAATTTGCCGCCATTTTCTTATCTTCTTTCCAGCAACCAAAACA 3360

DNA_HJX74	AAAAGATAACCTCCGGACTTTTGGAGTAATTTGCCGCCATTTTCTTATCTTCTTTCCAGCAACCAAAACA 3360

CDS_Nip	---------------------------------------------------------------------- 363

DNA_Nip	ATGAGAATTTTATGCTATAGAAATTTTCTCTTATTTTCATAGTTTGTGAAACATTGGCATGGGGAAGTTC 3430

DNA_NIL-K	ATGAGAATTTTATGCTATAGAAATTTTCTCTTATTTTCATAGTTTGTGAAACATTGGCATGGGGAAGTTC 3430

DNA_HJX74	ATGAGAATTTTATGCTATAGAAATTTTCTCTTATTTTCATAGTTTGTGAAACATTGGCATGGGGAAGTTC 3430

CDS_Nip	---------------------------------------------------------------------- 363

DNA_Nip	TTTGTGGTTACCAACTTGATTTATCCTAACAGAAAATAAAATATCTCAAGAAAGATTATATTATTTTGGA 3500

DNA_NIL-K	TTTGTGGTTACCAACTTGATTTATCCTAACAGAAAATAAAATATCTCAAGAAAGATTATATTATTTTGGA 3500

DNA_HJX74	TTTGTGGTTACCAACTTGATTTATCCTAACAGAAAATAAAATATCTCAAGAAAGATTATATTATTTTGGA 3500

CDS_Nip	----------------ACTCGGATCACAAAAGAGGACCTCTGCGATCATGATTGGGAATTCCGTTTCACT 417

DNA_Nip	TTGTTCTCTTGTTCAGACTCGGATCACAAAAGAGGACCTCTGCGATCATGATTGGGAATTCCGTTTCACT 3570

DNA_NIL-K	TTGTTCTCTTGTTCAGACTCGGATCACAAAAGAGGACCTCTGCGATCATGATTGGGAATTCCGTTTCACT 3570

DNA_HJX74	TTGTTCTCTTGTTCAGACTCGGATCACAAAAGAGGACCTCTGCGATCATGATTGGGAATTCCGTTTCACT 3570

CDS_Nip	ATA------------------------------------------------------------------- 421

DNA_Nip	ATAGTAAGTTTTCGCTAACCTGTCATCCAATCTGCATGGTTTGAGATATGTGTTTATATTAGCCGGCATA 3640

DNA_NIL-K	ATAGTAAGTTTTCGCTAACCTGTCATCCAATCTGCATGGTTTGAGATATGTGTTTATATTAGCCGGCATA 3640

DNA_HJX74	ATAGTAAGTTTTCGCTAACCTGTCATCCAATCTGCATGGTTTGAGATATGTGTTTATATTAGCCGGCATA 3640

CDS_Nip	---------------------------------------------------------------------- 421

DNA_Nip	TCTTAATGCTAGTGGCTGCTACTGCACAACTGACTTACTAGTTTTTTAGTTCAAATGGTTTTAGTCAACC 3710

DNA_NIL-K	TCTTAATGCTAGTGGCTGCTACTGCACAACTGACTTACTAGTTTTTTAGTTCAAATGGTTTTAGTCAACC 3710

DNA_HJX74	TCTTAATGCTAGTGGCTGCTACTGCACAACTGACTTACTAGTTTTTTAGTTCAAATGGTTTTAGTCAACC 3710

CDS_Nip	---------------------------------------------------------------------- 421

DNA_Nip	ACACCTGATGGATGCATGGGAATCATGAGTTCATGACAACTGTCAGCATCTGGTCTAGCTTCTTCAGATG 3780

DNA_NIL-K	ACACCTGATGGATGCATGGGAATCATGAGTTCATGACAACTGTCAGCATCTGGTCTAGCTTCTTCAGATG 3780

DNA_HJX74	ACACCTGATGGATGCATGGGAATCATGAGTTCATGACAACTGTCAGCATCTGGTCTAGCTTCTTCAGATG 3780

CDS_Nip	---------------------------------------------------------------------- 421

DNA_Nip	TCTTAGCTTCCAAATGCAATGAGGGAGAGTTGATGCATATCTCTTCGTGGTGGTAATTAAAGGTCGTAAA 3850

DNA_NIL-K	TCTTAGCTTCCAAATGCAATGAGGGAGAGTTGATGCATATCTCTTCGTGGTGGTAATTAAAGGTCGTAAA 3850

DNA_HJX74	TCTTAGCTTCCAAATGCAATGAGGGAGAGTTGATGCATATCTCTTCGTGGTGGTAATTAAAGGTCGTAAA 3850

CDS_Nip	---------------------------------------------------------------------- 421

DNA_Nip	CTTTGATTCATTGATTTGTCTGGTCAAGGATGATATTTGACTATTGATTTTTCTTGAGCATCACCTAAAA 3920

DNA_NIL-K	CTTTGATTCATTGATTTGTCTGGTCAAGGATGATATTTGACTATTGATTTTTCTTGAGCATCACCTAAAA 3920

DNA_HJX74	CTTTGATTCATTGATTTGTCTGGTCAAGGATGATATTTGACTATTGATTTTTCTTGAGCATCACCTAAAA 3920

CDS_Nip	---------------------------------------------------------------------- 421

DNA_Nip	CATATATACAAAAACTTTGAAAACCATACTTGTGTTCGTCTCTCTTACACTAAGCAACCGCATGATGATC 3990

DNA_NIL-K	CATATATACAAAAACTTTGAAAACCATACTTGTGTTCGTCTCTCTTACACTAAGCAACCGCATGATGATC 3990

DNA_HJX74	CATATATACAAAAACTTTGAAAACCATACTTGTGTTCGTCTCTCTTACACTAAGCAACCGCATGATGATC 3990

CDS_Nip	---------------------------------------------------------------------- 421

DNA_Nip	TGATGAATGAATTATTCATAGCATAAAATCTGTGATGTATCATGGCCATAAATTTAAATTCAACTCTCCA 4060

DNA_NIL-K	TGATGAATGAATTATTCATAGCATAAAATCTGTGATGTATCATGGCCATAAATTTAAATTCAACTCTCCA 4060

DNA_HJX74	TGATGAATGAATTATTCATAGCATAAAATCTGTGATGTATCATGGCCATAAATTTAAATTCAACTCTCCA 4060

CDS_Nip	---------------------------------------------------------------------- 421

DNA_Nip	CCATGTGCAGCATGCAGTTATCTGCTCAAAATTTGTAATTTGACGATATATTGTTAGGCATTACCAGAAA 4130

DNA_NIL-K	CCATGTGCAGCATGCAGTTATCTGCTCAAAATTTGTAATTTGACGATATATTGTTAGGCATTACCAGAAA 4130

DNA_HJX74	CCATGTGCAGCATGCAGTTATCTGCTCAAAATTTGTAATTTGACGATATATTGTTAGGCATTACCAGAAA 4130

CDS_Nip	---------------------------------------------------------------------- 421

DNA_Nip	AAAGGCTTAATAAGCCAACACATTTGTTTTAGTTTTTTTAGTAAGAACTAAATCTGCTTTGCAAATGAAA 4200

DNA_NIL-K	AAAGGCTTAATAAGCCAACACATTTGTTTTAGTTTTTTTAGTAAGAACTAAATCTGCTTTGCAAATGAAA 4200

DNA_HJX74	AAAGGCTTAATAAGCCAACACATTTGTTTTAGTTTTTTTAGTAAGAACTAAATCTGCTTTGCAAATGAAA 4200

CDS_Nip	---------------------------------------------------------------------- 421

DNA_Nip	TGTACAATTATACTCCAGATGAGTAATACCAAAATATAACCATTCTACACTTTTTGTAATTATATGATTA 4270

DNA_NIL-K	TGTACAATTATACTCCAGATGAGTAATACCAAAATATAACCATTCTACACTTTTTGTAATTATATGATTA 4270

DNA_HJX74	TGTACAATTATACTCCAGATGAGTAATACCAAAATATAACCATTCTACACTTTTTGTAATTATATGATTA 4270

CDS_Nip	----------------------------------------GCAGCACCAGAATATTGGAGAAACCTGGAT 450

DNA_Nip	ATCATGATGTTCATAATTTCTGTTTCCTTTGCCCGTGCAGGCAGCACCAGAATATTGGAGAAACCTGGAT 4340

DNA_NIL-K	ATCATGATGTTCATAATTTCTGTTTCCTTTGCCCGTGCAGGCAGCACCAGAATATTGGAGAAACCTGGAT 4340

DNA_HJX74	ATCATGATGTTCATAATTTCTGTTTCCTTTGCCCGTGCAGGCAGCACCAGAATATTGGAGAAACCTGGAT 4340

CDS_Nip	CCATCATGGAAACATACCGGTCCACCAATGCGACGTTACTTCCACCCTGATGGTTACCACAGCGCAGATC 520

DNA_Nip	CCATCATGGAAACATACCGGTCCACCAATGCGACGTTACTTCCACCCTGATGGTTACCACAGCGCAGATC 4410

DNA_NIL-K	CCATCATGGAAACATACCGGTCCACCAATGCGACGTTACTTCCACCCTGATGGTTACCACAGCGCAGATC 4410

DNA_HJX74	CCATCACGGAAACATACCGGTCCACCAATGCGACGTTACTTCCACCCTGATGGTTACCACAGCGCAGATC 4410

CDS_Nip	CTCATGATGCTGTATGGGGTGGCCATGAGTGCACGTACACGATCATAACAAGCTTTGCTGGTAATGGATG 590

DNA_Nip	CTCATGATGCTGTATGGGGTGGCCATGAGTGCACGTACACGATCATAACAAGCTTTGCTGGTAATGGATG 4480

DNA_NIL-K	CTCATGATGCTGTATGGGGTGGCCATGAGTGCACGTACACGATCATAACAAGCTTTGCTGGTAATGGATG 4480

DNA_HJX74	CTCATGATGCTGTATGGGGTGGCCATGAGTGCACGTACACGATCATAACAAGCTTTGCTGGTAATGGATG 4480

CDS_Nip	TATCAGGGATCATTATGTGAGGATCAATCGGTGGCCACCGATGAAAGTTTCAAGGAAGGAGGATTGGAGC 660

DNA_Nip	TATCAGGGATCATTATGTGAGGATCAATCGGTGGCCACCGATGAAAGTTTCAAGGAAGGAGGATTGGAGC 4550

DNA_NIL-K	TATCAGGGATCATTATGTGAGGATCAATCGGTGGCCACCGATGAAAGTTTCAAGGAAGGAGGATTGGAGC 4550

DNA_HJX74	TATCAGGGATCATTATGTGAGGATCAATCGGTGGCCACCGATGAAAGTTTCAAGGAAGGAGGATTGGAGC 4550

CDS_Nip	TGGGAGCTATCCAACCATCTTTACCGCTACAACAGCATCCCTGACACTGACAAGAAGGGATGTACAGGTC 730

DNA_Nip	TGGGAGCTATCCAACCATCTTTACCGCTACAACAGCATCCCTGACACTGACAAGAAGGGATGTACAGGTC 4620

DNA_NIL-K	TGGGAGCTATCCAACCATCTTTACCGCTACAACAGCATCCCTGACACTGACAAGAAGGGATGTACAGGTC 4620

DNA_HJX74	TGGGAGCTATCCAACCATCTTTACCGCTACAACAGCATCCCTGACACTGACAAGAAGGGATGTACAGGTC 4620

CDS_Nip	CTCTGTTCCCCGTTTGGTGA-------------------------------------------------- 750

DNA_Nip	CTCTGTTCCCCGTTTGGTGAATGAATCCACTTCTATGGTAGTAGCAGTGACCGGTAATAGTCGCGATCTC 4690

DNA_NIL-K	CTCTGTTCCCCGTTTGGTGAATGAATCCACTTCTATGGTAGTAGCAGTGACCGGTAATAGTCGCGATCTC 4690

DNA_HJX74	CTCTGTTCCCCGTTTGGTGAATGAATCCACTTCTATGGTAGTAGCAGTGACCGGTAATAGTCGCGATCTC 4690

CDS_Nip	---------------------------------------------------------------------- 750

DNA_Nip	CAACTCATAGAAACTGGCCTCGGATCGTTCCTGACCCTGAGGAACGTGATAACGAGAGGCTCTGTTTGGC 4760

DNA_NIL-K	CAACTCATAGAAACTGGCCTCGGATCGTTCCTGACCCTGAGGAACGTGATAACGAGAGGCTCTGTTTGGC 4760

DNA_HJX74	CAACTCATAGAAACTGGCCTCGGATCGTTCCTGACCCTGAGGAACGTGATAACGAGAGGCTCTGTTTGGC 4760

CDS_Nip	---------------------------------------------------------------------- 750

DNA_Nip	AGTTATGAGCTATGAACACCAATGGAGATTAAGCTTACTTATGTGGTATACTGGTATGTAACGTACTCCT 4830

DNA_NIL-K	AGTTATGAGCTATGAACACCAATGGAGATTAAGCTTACTTATGTGGTATACTGGTATGTAACGTACTCCT 4830

DNA_HJX74	AGTTATGAGCTATGAACACCAATGGAGATTAAGCTTACTTATGTGGTATACTGGTATGTAACGTACTCCT 4830

CDS_Nip	------------------------------------------------------------------- 750

DNA_Nip	ATATAGTACTACATGATTTCCTGTGCGTTGCTGCTGGAATTGGTATAAAACTTATGTTTGCAAATTT 4897

DNA_NIL-K	ATATAGTACTACATGATTTCCTGTGCGTTGCTGCTGGAATTGGTATAAAACTTATGTTTGCAAATTT 4897

DNA_HJX74	ATATAGTACTACATGATTTCCTGTGCGTTGCTGCTGGAATTGGTATAAAACTTATGTTTGCAAATTT 4897

**Fig. S2** **DNA sequence alignment of *SDEF* between HJX74 and NIL-K.** UTRs were shaded in green, exons in black, introns in grey, and the single-nucleotide substitution in red. Nip, Nipponbare.

**Fig. S3** **Temporal and spatial expression profiles of candidate genes in *qSD7-1*.** **a**, A heat map of the relative expression of nine candidate genes. All expression data were prepared by the web tool RiceXPro (http://ricexpro.dna.affrc.go.jp/). The grey boxes mean no expression data. The heat map was made by TBtools (v2.016). **b**, The relative expression of nine candidate genes by qRT-PCR. *OsUBC* (*LOC_Os02g42314*) was used as a reference gene. Data are means ± SD (n = three biological replicates). * *P* < 0.05, ** *P* < 0.01, Student’s *t*-test. ns, not significant. DAP, days after pollination.

**Fig. S4 Comparison of the major agronomic traits of HJX74, NIL-K, and NIL-J. a-g**, Comparison of plant height (**a**), effective panicle number (**b**), grain number per panicle (**c**), grain length (**d**), grain width (**e**), 1000-grains weight (**f**), and yield per plant (**g**) between HJX74, NIL-K and NIL-J. Data are means ± SD. *n* = 58 (**a**), 15 (**b**), 10 (**c**), 5 (**d**), 5 (**e**), 15 (**f**), 10 (**g**) plants. * *P* < 0.05, ** *P* < 0.01, *** *P* < 0.001, **** *P* < 0.0001. ns, not significant. Student’s *t*-test.

**Fig. S5 F-box domain analysis of SDEF. a**, Schematic structure of the SDEF protein. The red rectangle shows the F-box like domain (FBLD). **b**, F-box domain alignment of SDEF and its homologs. Identical amino acids are shaded in black and conserved changes in red. Homologs are from *Panicum virgatum* (XP_039784084.1), *Setaria italica* (XP_004982956.1), *Triticum aestivum* (XP_044395551.1), *Hordeum vulgare* (XP_044970079.1), *Aegilops tauschii* (XP_020165376.1), *Brachypodium distachyon* (XP_003574011.1), *Zea mays* (ACG42305.1), and *Sorghum bicolor* (XP_002467126.1).

**Fig. S6 Protein sequence multiple alignment of SDEF with its homologs.** Identical amino acids are shaded in black and conserved changes in gray. The conserved F-box-like domain is underlined. The tryptophan (Trp, W) at position 153, a natural variation between NIL-K and HJX74, is indicated by an asterisk. All sequences were obtained from the NCBI GenBank database. Accession numbers and species are indicated as follows: XP_039784084.1 (*Panicum virgatum*), XP_004982956.1 (*Setaria italica*), XP_044395551.1 (*Triticum aestivum*), XP_044970079.1 (*Hordeum vulgare*), XP_020165376.1 (*Aegilops tauschii*), XP_003574011.1 (*Brachypodium distachyon*), ACG42305.1 (*Zea mays*), and XP_002467126.1 (*Sorghum bicolor*).

**Fig. S7** **Phylogenetic tree of SDEF and other F-box proteins in flowering plants.** Bootstrap values (1,000 replicates) are shown at nodes. SDEF is highlighted in red.

**Fig. S8** ***pSDEF::GUS* staining in young root (a, b), reproductive stem (c) and young leaf (d, e).** Cross sections for root (**b**) and leaf (**e**) are shown. Scale bars, 1 mm (**a**, **d**), 2 mm (**c**) and 50 μm (**b**, **e**).

**Fig. S9** **Y2H assays showing the interactions of Culling with SDEF and OSK1.** Yeast transformants were grown on control SD-WL (-Trp/-Leu) or selective SD-WLHA (-Trp/-Leu/-His/-Ade) media.

**Fig. S10 Comparison of ABA (a) and IAA (b) content in the developing seeds between HJX74 and NIL-K.** FW, fresh weight. Data are means ± SD (n = three biological replicates). * *P* < 0.05, ** *P* < 0.01, *** *P* < 0.001, **** *P* < 0.0001. ns, not significant. Student’s *t*-test.

**Fig. S11** **Germination percentages of freshly harvested panicles in different rice subpopulations.** Data are means ± SE after 7 d imbibition. *n* = 4 (IND I), 12 (IND II), 25 (IND III), 9 (IND INT), 3 (AUS), 3 (INT), 3 (TEJ) and 2 (TRJ) accessions. IND I, *indica* I; IND II, *indica* II; IND III, *indica* III; IND INT, *indica* intermediate; AUS, *aus*; INT, intermediate; TEJ, temperate *japonica*; TRJ, tropical *japonica.*

**Fig. S12 Subpopulation-matched comparison of seed dormancy among Hap2, Hap3 and Hap5.** Germination rates of freshly harvested panicles were compared within the same IND III and IND INT subpopulations. Data are means ± SE after 7 d imbibition. *n* = 13 (Hap2), 6 (Hap3) and 13 (Hap5) accessions. Each accession contains ten independent plants. Different letters denote significant differences (*P* < 0.05, Duncan’s multiple-range test).

**Fig. S13 Phenotypic comparison of seed dormancy between Hap1 (457-T) and the rare Hap (457-C) accessions.** Data are means ± SE after 7 d imbibition. *n* = 20 (Hap1, 457-T) and 10 (rare Hap, 457-C) accessions. Each accession contains ten independent plants. Student’s *t*-test. ns, not significant.
